# Nonoptimum Temperatures Are More Closely Associated With Fatal Myocardial Infarction Than With Nonfatal Events

**DOI:** 10.1016/j.cjca.2023.08.026

**Published:** 2023-12

**Authors:** Piaopiao Hu, Jie Chang, Yulin Huang, Moning Guo, Feng Lu, Ying Long, Huan Liu, Xudong Yang, Yue Qi, Jiayi Sun, Zhao Yang, Qiuju Deng, Jing Liu

**Affiliations:** a Center for Clinical and Epidemiologic Research, Beijing An Zhen Hospital, Capital Medical University, Beijing Institute of Heart, Lung, and Blood Vessel Diseases, Beijing, China; b National Clinical Research Center of Cardiovascular Diseases, Beijing, China; c Key Laboratory of Remodelling-Related Cardiovascular Diseases, Ministry of Education, Beijing, China; d Beijing Municipal Key Laboratory of Clinical Epidemiology, Beijing, China; e Beijing Municipal Health Big Data and Policy Research Center, Beijing, China; f Beijing Institute of Hospital Management, Beijing, China; g School of Architecture, Tsinghua University, Beijing, China; h State Key Joint Laboratory of ESPC, State Environmental Protection Key Laboratory of Sources and Control of Air Pollution Complex, School of Environment, Tsinghua University, Beijing, China; i Departments of Building Science and Vanke School of Public Health, Tsinghua University, Beijing, China

## Abstract

**Background:**

Ambient temperatures trigger hospitalisation, mortality, and emergency department visits for myocardial infarction (MI). However, nonoptimum temperature-related risks of fatal and nonfatal MI have not yet been compared.

**Methods:**

From 2007 to 2019, 416,894 MI events (233,071 fatal and 183,823 nonfatal) were identified in Beijing, China. A time-series analysis with a distributed-lag nonlinear model was used to compare the relative and population-attributable risks of fatal and nonfatal MI associated with nonoptimum temperatures.

**Results:**

The reference was the optimum temperature of 24.3°C. For single-lag effects, cold (−5.2°C) and heat (29.6°C) effects had associations that persisted for more days for fatal MI than for nonfatal MI. For cumulative-lag effects over 0 to 21 days, cold effects were higher for fatal MI (relative risk [RR] 1.99, 95% confidence interval [CI] 1.68-2.35) than for nonfatal MI (RR 1.60, 95% CI 1.32-1.94) with a *P* value for difference in effect sizes of 0.048. In addition, heat effects were higher for fatal MI (RR 1.33, 95% CI 1.24-1.44) than for nonfatal MI (RR 0.99, 95% CI 0.91-1.08) with a *P* value for difference in effect sizes of 0.002. The attributable fraction of nonoptimum temperatures was higher for fatal MI (25.6%, 95% CI 19.7%-30.6%) than for nonfatal MI (19.1%, 95% CI 12.1%-25.0%).

**Conclusions:**

Fatal MI was more closely associated with nonoptimum temperatures than nonfatal MI, as evidenced by single-lag effects that have associations which persisted for more days, higher cumulative-lag effects, and higher attributable risks for fatal MI. Strategies are needed to mitigate the adverse effects of nonoptimum temperatures.

## Graphical abstract

**Figure:**
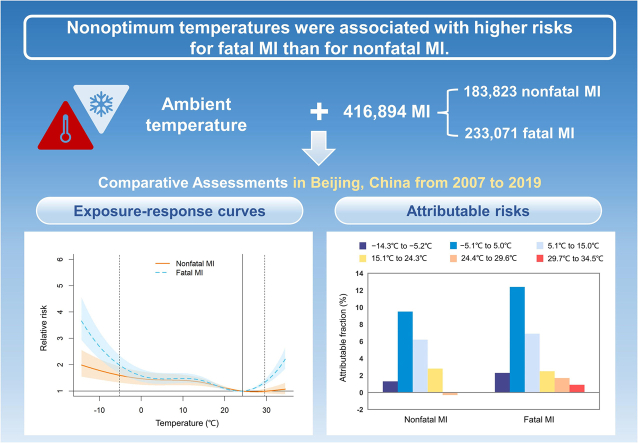

## Résumé

**Contexte:**

Certaines températures ambiantes entraînent des hospitalisations, de la mortalité et des consultations aux urgences en cas d’infarctus du myocarde (IM). Cependant, les risques d’IM mortel et non mortel liés à des températures non optimales n’ont pas encore fait l’objet d’une comparaison.

**Méthodologie:**

De 2007 à 2019, 416 894 IM (233 071 mortels et 183 823 non mortels) ont été signalés à Beijing, en Chine. Une analyse des séries chronologiques faisant appel à un modèle non linéaire d’effets retardés échelonnés a été utilisée pour comparer les risques relatifs et populationnels d’IM mortels et non mortels associés à des températures non optimales.

**Résultats:**

La référence était la température optimale de 24,3 °C. Dans le cas des effets retardés simples, les températures froides (−5,2 °C) et chaudes (29,6 °C) affichaient des associations qui persistaient durant plus de jours pour l’IM mortel que pour l’IM non mortel. Pour ce qui est des effets retardés cumulatifs sur 0 à 21 jours, les effets du froid ont été plus marqués dans le cas des IM mortels (risque relatif [RR] de 1,99; intervalle de confiance [IC] à 95 % : 1,68-2,35) que dans le cas des IM non mortels (RR de 1,60; IC à 95 % : 1,32-1,94) avec une valeur *p* de 0,048 pour la différence quant à l’amplitude des effets. De plus, les effets de la chaleur ont été plus marqués dans le cas des IM mortels (RR de 1,33; IC à 95 % : 1,24-1,44) que dans le cas des IM non mortels (RR de 0,99; IC à 95 % : 0,91-1,08) avec une valeur *p* de 0,002 pour la différence quant à l’amplitude des effets. La fraction attribuable aux températures non optimales était plus élevée pour les IM mortels (25,6 %; IC à 95 % : 19,7 %-30,6 %) que pour les IM non mortels (19,1 %; IC à 95 % : 12,1 %-25,0 %).

**Conclusions:**

L’IM mortel a été plus étroitement associé à des températures non optimales que ne l’a été l’IM non mortel, comme l’ont démontré les effets retardés simples dont les associations ont persisté durant un plus grand nombre de jours, les effets retardés cumulatifs plus marqués et les risques attribuables plus marqués pour les IM mortels. Des stratégies sont donc nécessaires pour atténuer les effets indésirables associés des températures non optimales.

Ischemic heart disease is a leading threat to public health and an increasing global burden.^1^ Myocardial infarction (MI), the most severe manifestation of ischemic heart disease, can be triggered by environmental factors, including frequent and intense extreme temperatures.^2^ Thus, quantifying the deleterious effects of temperature on MI is essential for developing public health policies and early warning systems.

Studies have found that extreme cold and heat temperatures may trigger hospitalisations,^3^ mortality,^4^ and emergency department visits for MI,^5^ but whether the effects of nonoptimum temperatures differ in nonfatal MI and fatal MI (including in-hospital and out-of-hospital deaths) has not been well evaluated quantitatively.^6^, ^7^, ^8^, ^9^ Only 4 studies from Europe reported the relative risks (RRs) of temperature-related fatal and nonfatal MI separately but lacked comparative assessments and statistical tests for the differences.^6^, ^7^, ^8^, ^9^ The inconsistency of their findings was shown by the RRs. In one study, the cold effect for fatal MI was slightly higher than that of nonfatal MI, and conversely, the heat effect for nonfatal MI was slightly higher than that of fatal MI.^6^ However, there was little difference in temperature-related risk of fatal and nonfatal MI in other studies.^7^, ^8^, ^9^ This discrepancy between studies is probably due to the limited sample sizes and failure to adjust for air pollutants, which are closely related to cardiovascular health and ambient temperatures.^10^ In addition, previous studies rarely considered the lag effect or used only short time windows of up to 10 days.^6^ More importantly, the impact of ambient temperature on fatal and nonfatal MI has not been assessed from the perspective of public health. Such an assessment of population-attributable risk is necessary to quantify the potential impact of control measures on the population.^11^

Based on the findings of the studies mentioned above, we hypothesised that MI morbidity is associated with ambient temperatures and that this association differs between fatal and nonfatal MI. We aimed to test this hypothesis through a 13-year time-series study using data from 2007 to 2019 in the Beijing Cardiovascular Disease Surveillance System (BCDSS).

## Methods

### Study setting

Beijing, the capital of China, is located in the Northern China Plain (39°56′N and 116°20′E) with an area of 16,410 km^2^. It has a typical continental monsoon climate, characterised by hot and rainy summers, cold and dry winters, and short springs and autumns.^12^ The annual temperatures range from −20 to 40°C, with an average of 11 to 13°C.^13^

### Study design

The time-series design uses ambient temperature and counts of MI events collected at ordered and daily time intervals and controls for time-varying confounders (relative humidity, particulate matter ≤ 2.5 μm [PM_2.5_], and day of the week) and slowly varying confounders (a smooth time function to adjust for seasonal and long-term trends), thus enabling assessment of the association between temperature and MI events and further the delayed (“lagged”) association, including the effects at different single lags after the exposure (single-lag effect), along with cumulative lags after the exposures experienced over multiple days (cumulative-lag effect).^14^^,^^15^

### Study population

Our institution served as the centre of the World Health Organisation Monitoring Trends and Determinants in Cardiovascular Disease (WHO MONICA) project in China from 1984 to 1993.^16^ After accomplishment of the WHO MONICA project, we established the BCDSS using personal identification information by linking records routinely collected in the Beijing Hospital Discharge Information System (HDIS) and Beijing Vital Registration Monitoring System (VRMS).^17^

MI morbidity refers to the total MI events, including nonfatal or fatal events.^6^ Nonfatal events were identified by the International Classification of Diseases, Tenth Revision (ICD-10) codes I21 to I22 (acute MI and subsequent MI) in the principal discharge diagnoses, which refer to MI events in patients who survive for at least 28 days after hospital admission. Fatal events were identified by ICD-10 codes I20 to I25 for the underlying causes of death and were further classified as in-hospital or out-of-hospital deaths based on the site of death. The same deaths identified in both HDIS and VRMS were linked to 1 case. We had previously validated the diagnosis of MI in the BCDSS.^18^ The positive and negative predictive values of an acute MI diagnosis in the HDIS compared with the WHO MONICA criteria were 94.4% and 96.1%, respectively.

The system identified 435,775 MI events among permanent residents aged ≥ 35 years from January 1, 2007, to December, 31, 2019. Multiple steps were taken to avoid repeatedly counting MI events.^19^ The patients were deemed to have experienced a sequential course of care if rehospitalised or transferred on the day after discharge (n = 7649). Patients were deemed unlikely to have had an acute MI and were excluded if they were discharged alive with a duration of hospitalisation of less than 1 day and were not rehospitalised or died on that day (n = 3714). Patients with a record of any subsequent rehospitalisation or death within 28 days (n = 7516) were considered to have had 1 MI event according to the WHO MONICA protocols, and the onset time of the first event and the outcome within 28 days were retained. Information on sex and age was obtained for each MI event, and 2 records with missing values for sex were excluded. In total, 416,894 MI events were included (Supplemental Fig. S1).

The characteristics of the MI events were further identified. Types of MI were identified according to the principal discharge diagnoses using ICD-10 codes I21.0 to I21.3, I22.0, I22.1, and I22.8 for ST-segment elevation myocardial infarction (STEMI), and I21.4 for non-ST-segment elevation myocardial infarction (NSTEMI). Comorbidities were identified according to secondary discharge diagnoses (up to 7 diagnoses) using ICD-10 codes I46 to I49 for arrhythmia, I50 for heart failure, J44 to J45 for chronic obstructive pulmonary disease, I10 to I15 for hypertension, and E10 to E14 for diabetes. Payment methods (urban health insurance or public reimbursement/other) were used to represent socioeconomic status. Reperfusion therapy was identified according to International Classification of Diseases, 9th Revision, Clinical Modification Operations and Procedures codes 00.66, 36.01, 36.02, 36.05, 36.06, and 36.07 for percutaneous coronary intervention and 36.1 for coronary artery bypass grafting.

### Environmental data

From January 1, 2007, to December 31, 2019, daily outdoor mean temperature (°C) and mean relative humidity (%) data were obtained from the China Meteorologic Data Sharing Service System (http://data.cma.cn). Data of 10-km spatial resolution PM_2.5_ concentrations (μg/m^3^) were derived from Tracking Air Pollution in China (http://tapdata.org.cn).^20^, ^21^, ^22^, ^23^ We then averaged these estimates to obtain daily exposures at the city level.

### Statistical analyses

Daily MI events, demographic characteristics of all patients with MI by sex and age groups, daily meteorologic factors (daily mean temperature and relative humidity), and PM_2.5_ were summarised as mean, SD, minimum, maximum, and percentile.

Temperature-related associations with MI events were analysed with the use of distributed-lag nonlinear models (DLNMs) with negative binomial distributions for the outcomes, controlling for relative humidity, PM_2.5_, day of the week, and time (seasonal and long-term trends).^24^^,^^25^ DLNM can flexibly explore the effects that vary simultaneously along with the dimension of exposure and lag to obtain single-lag effects and cumulative-lag effects. We used 2 natural cubic spline functions with 4 degrees of freedom (*df*) and 3 internal knots for the cross-basis of the exposure response and the lag response. The lag period was extended to 21 days to consider the long delay of the cold and harvesting effects (temperature-vulnerable populations led to an initial rapid increase in morbidity with a subsequent decrease). The model was expressed with the following equation:

$$\text{logE}\left[Y_{t}\right]=\alpha +\beta T_{t,l}+ns\left({Relative\;humidity}_{t},df=3\right)+ns\left({PM2.5}_{t},df=3\right)+ns\left(Time,7\;\text{df}*\text{years}\right)+{Dow}_{t}\text{,}$$

where T_t,l_ is a 2-dimensional cross-basis matrix, l is the maximum number of lag days, time is used to adjust for seasonal and long-term trends, Dow_t_ is the day of the week on day t, and ns() is the natural cubic spline. Referring to previous studies, we used 3 *df*s for relative humidity and PM_2.5_ and 7 *df*s per year for time.

Referring to the previous method,^26^ we defined the optimum temperature as the temperature corresponding to the lowest risk, that is, minimum morbidity temperature (MMT), which was 24.3°C in this study. For single-lag association, we depicted a 3-dimensional plot of RRs of MI along with the full temperatures and 21 lag days as well as an overall lag structure figure for extreme cold (−5.2°C, 2.5th percentile) and heat (29.6°C, 97.5th percentile). For the cumulative-lag association, we plotted the cumulative exposure-response curves over 0 to 21 lag days for associations between daily temperatures and MI events, and defined the cold and heat effects as the cumulative-lag risks over 0 to 21 lag days at extreme cold (−5.2°C) and extreme heat (29.6°C) relative to the MMT. Stationary block bootstrap based on a block length of 10 by 1000 times samples was used to characterise statistically significant differences in RR between fatal and nonfatal MI and between in-hospital fatal and out-of-hospital fatal MI.^27^ *P* < 0.05 indicated a significant difference in effect sizes.

For the burden of MI related to nonoptimum temperatures, we calculated the attributable fraction (AF) by a backward method.^28^ We used the 2.5th percentile (−5.2°C), MMT (24.3°C), and 97.5th percentile (29.6°C) as cutoff values to classify temperatures into extreme cold (−14.3 to −5.2°C), moderate cold (−5.1 to 24.3°C), moderate heat (24.4 to 29.6°C), and extreme heat (29.7 to 34.5°C).^26^ According to the district heating policy in Beijing, heating service is provided if the daily mean temperature is < 5°C for 5 consecutive days.^29^ To assess the specific temperature range in relation to the potential interest of the policy, moderate cold was further divided into 3 sections: −5.1 to 5.0°C, 5.1 to 15.0°C, and 15.1 to 24.3°C. Monte Carlo simulations were run 5000 times to obtain 95% empirical CIs.^28^

Subgroup analyses were performed by sex and age group (< 65 and ≥ 65 years, the latter further refined as 65-74, 75-84, and ≥ 85 years) in separate models. The *z* test was used to test the difference between the 2 RRs among subgroups with the following formula:

$$({\hat{E}}_{1}-{\hat{E}}_{2})∕\sqrt{{\left(S{\hat{E}}_{1}\right)}^{2}+{\left(S{\hat{E}}_{2}\right)}^{2}}$$

where Ȇ_1_ and Ȇ_2_ are the natural logarithms of RRs, and SȆ_1_ and SȆ_2_ are their respective standard errors.^30^ *P* < 0.05 indicated a significant interaction.

Sensitivity analyses were performed as follows. 1) We tested the model and considered different parameters, including overdispersion tests, *df* for cross-basis based on the Akaike information criterion,^25^ *df* for time (6, 8, and 10), and unadjusted and confounder-adjusted models. 2) We calculated the cold and heat effects, including changes in the maximum lag period (4, 14, 28, and 31 days), temperature cutoffs (first/99th percentiles, 5th/95th percentiles, and 10th/90th percentiles), and reference values (25th and 75th percentiles). 3) The onset time of the last event within 28 days was used instead of the first event. 4) We changed the block length (5 and 15) in the stationary block bootstrap to check whether the results were sensitive to the block length. 5) We performed subgroup analyses (types of MI, payment methods, comorbidities, and reperfusion therapy) to examine potential effect modifications. 6) We performed interactive and stratified analyses of temperature and PM_2.5_ on MI.

Statistical analyses were implemented using the R software (www.r-project.org) with the DLNM fitted using the “dlnm” package. All statistical tests were 2 tailed, and *P* < 0.05 was considered to be statistically significant.

## Results

### Descriptive statistics

There were 416,894 MI events (233,071 fatal and 183,823 nonfatal events) that occurred over 4748 days from 2007 to 2019. The patients’ mean age was 72.2 ± 13.5 years, and 60.1% were men (Supplemental Table S1). There were 87.8 MI cases per day on average (range 31 to 169), of which 38.7 were nonfatal and 49.1 were fatal (in-hospital: 20.0 events; out-of-hospital: 29.1 events). Daily mean temperature averaged 13.6°C with a range of −14.3 to 34.5°C over the 13 years. The mean relative humidity was 51.4 ± 20.1% and mean PM_2.5_ concentration was 73.2 ± 46.9 μg/m^3^ (Table 1). Apparent seasonal patterns and long-term trends in daily MI events were observed in the time-series plots, and relatively more events occurred during the cold months from November to January (Supplemental Fig. S2).

**Table 1:** Descriptive statistics of daily myocardial infarction events, meteorologic factors, and PM_2.5_ concentrations in Beijing, China, 2007-2019

	Mean	SD	Min	25th	50th	75th	Max
Total MI, n	87.8	22.4	31.0	71.0	87.0	103.0	169.0
Nonfatal MI	38.7	12.4	0	30.0	38.0	47.0	85.0
Fatal MI	49.1	12.9	14.0	40.0	48.0	57.0	114.0
In-hospital fatal MI	20.0	6.5	3.0	15.0	19.0	24.0	55.0
Out-of-hospital fatal MI	29.1	8.5	5.0	23.0	29.0	35.0	87.0
Total MI by characteristics, n							
Men	52.8	14.2	14.0	43.0	52.0	62.0	110.0
Women	35.0	10.1	8.0	28.0	34.0	42.0	84.0
< 65 years old	25.3	7.3	4.0	20.0	25.0	30.0	54.0
≥ 65 years old	62.5	17.2	18.0	50.0	61.0	74.0	130.0
Daily meteorology and air pollution							
Mean temperature, °C	13.6	11.3	−14.3	2.7	15.1	24.1	34.5
Relative humidity, %	51.4	20.1	8.0	35.0	52.0	68.0	99.0
PM_2.5_, μg/m^3^	73.2	46.9	3.8	37.7	62.9	96.6	359.6

25th, 25th percentile; 50th, 50th percentile; 75th, 75th percentile; Max, maximum; MI, myocardial infarction; Min, minimum; PM_2.5_, particulate matter ≤ 2.5 μm in aerodynamic diameter.

### Associations between ambient temperatures and MI

**Figure 1:**
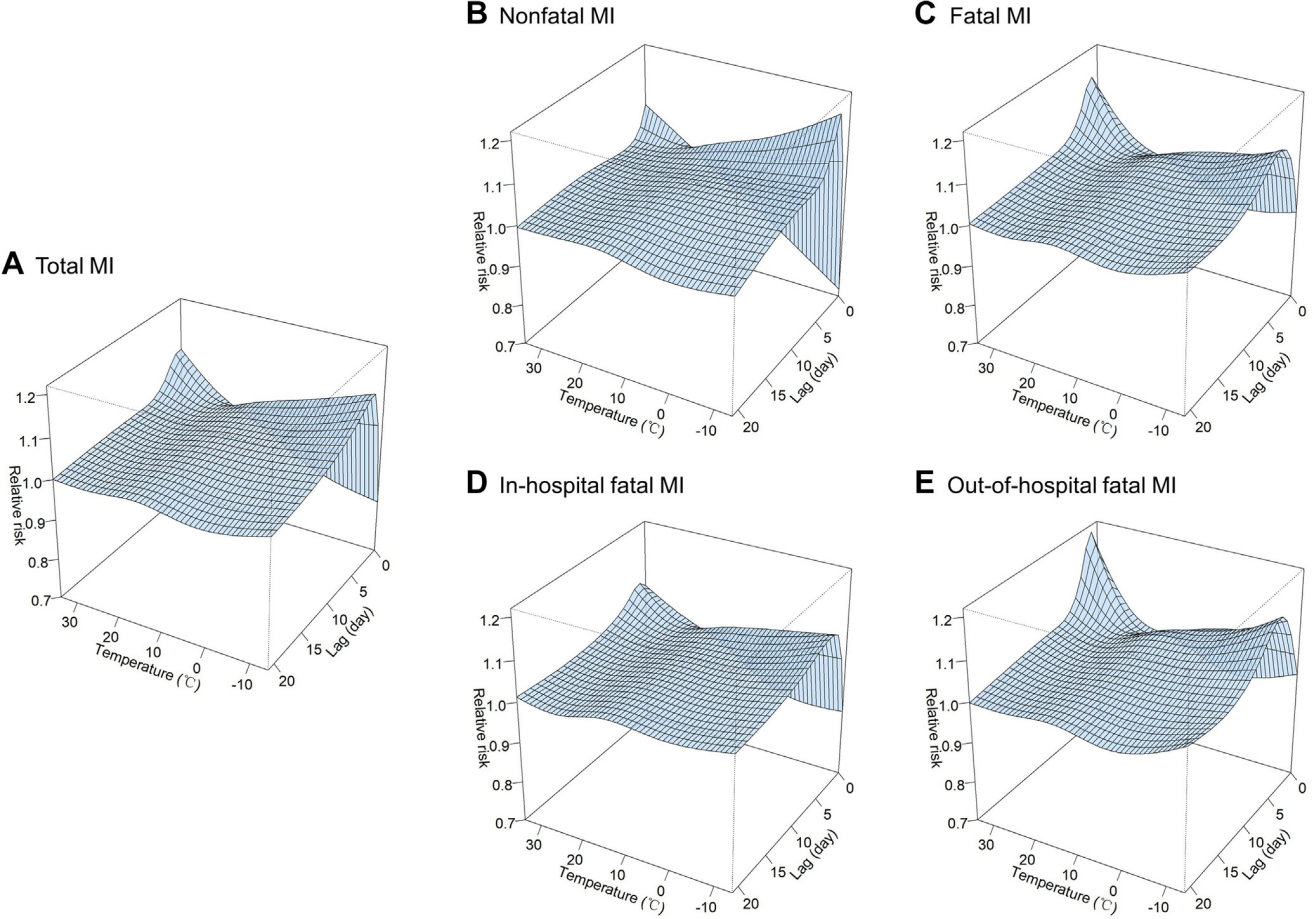
Three-dimensional plots of relative risks of myocardial infarction (MI) along with temperatures and lag days.

**Figure 2:**
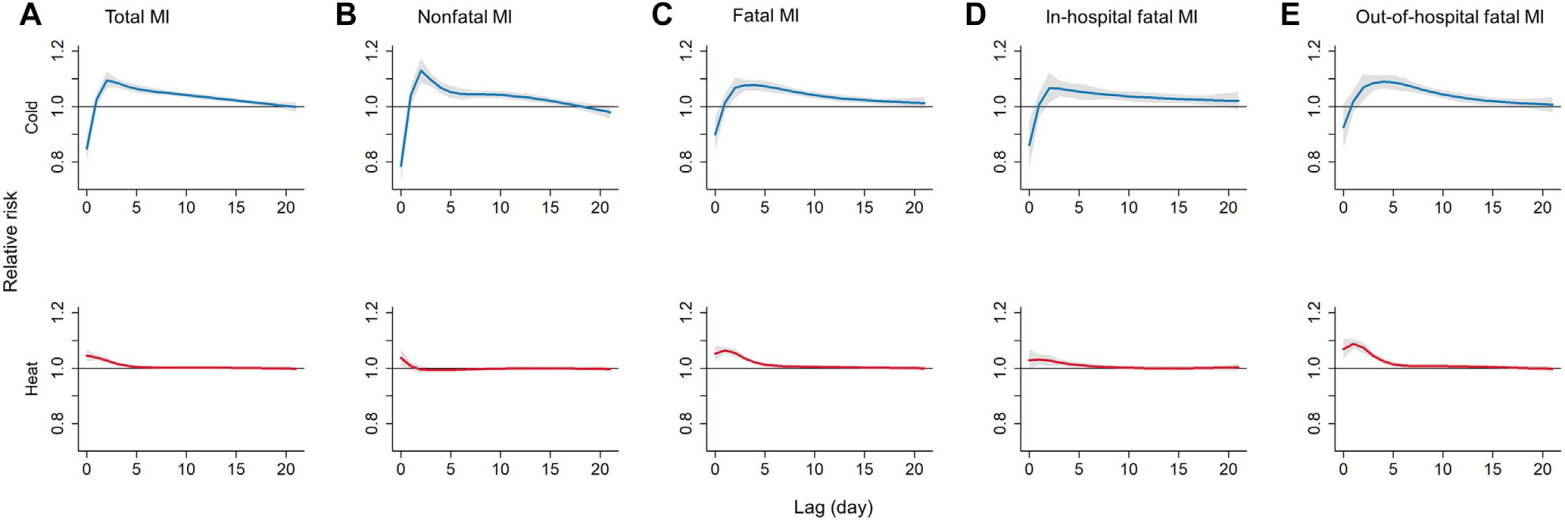
Overall lag structure in relative risks of extreme temperatures on myocardial infarction (MI) events. The blue and red lines show the mean estimates of extreme cold (−5.2°C, 2.5th percentile) and heat (29.6°C, 97.5th percentile) temperature-related risk, respectively; the shaded areas show 95% confidence intervals.

For the single-lag association, the 3-dimensional plot suggested a nonlinear association between temperature and MI along with a 21-day lag (Fig. 1). For MI morbidity, the effects of extreme cold occurred on lag day 2 and then decreased on subsequent days until the effect disappeared on lag day 17. In contrast, the effects of extreme heat occurred on the day and were then drastically attenuated to lag day 3. The effects had associations that persisted for more days for fatal MI than for nonfatal MI in extreme cold (lag day 17 vs lag day 15) and heat (lag day 5 vs the day) (Fig. 2; Supplemental Tables S2 and S3).

**Figure 3:**
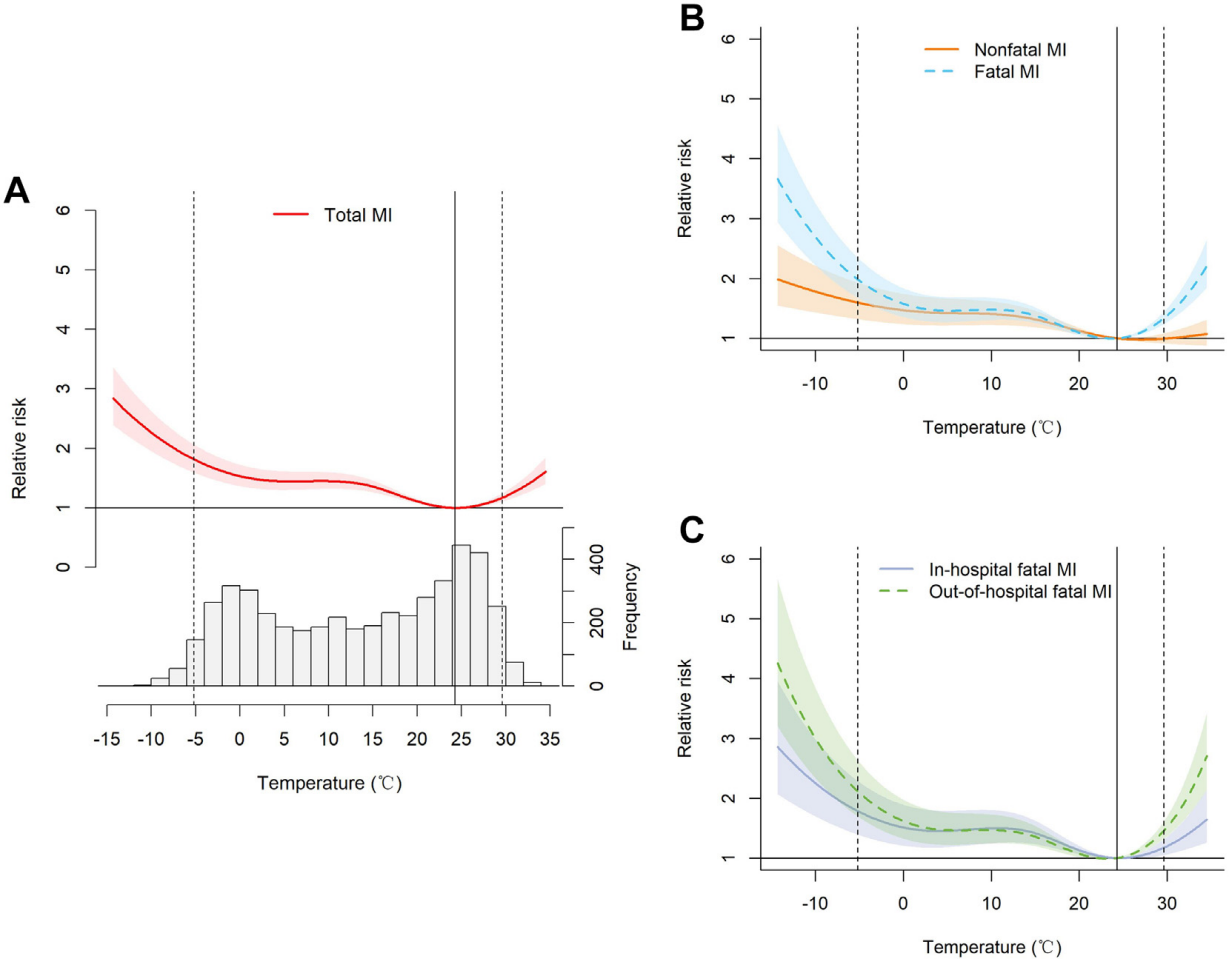
Cumulative exposure-response curves over 0 to 21 lag days for associations between daily mean temperature and myocardial infarction (MI) events. (**A**) Total MI, (**B**) nonfatal and fatal MI, (**C**) in-hospital and out-of-hospital fatal MI. The **black dotted lines** indicate extreme cold (−5.2°C, 2.5th percentile) and heat (29.6°C, 97.5th percentile) temperatures. The **black solid lines** indicate the minimum morbidity temperature (24.3°C).

For the overall cumulative association over 0 to 21 days, the exposure-response curve between temperature and MI events showed a nonlinear and inverse J-shaped association, and the risk was elevated when temperatures were above or below MMT of 24.3°C. Compared with nonfatal MI, a more prominent and substantially increased magnitude of the risks related to nonoptimum temperatures was observed in fatal MI when the temperature was lower than ∼ 0°C or higher than that of MMT (Fig. 3).

The cold and heat effects (RRs) of total MI were 1.81 (95% CI 1.59-2.06) and 1.17 (95% CI 1.10-1.24), respectively. Cold effects were higher for fatal MI (RR 1.99, 95% CI 1.68-2.35) than for nonfatal MI (RR 1.60, 95% CI 1.32-1.94) with a *P* value for difference in effect sizes of 0.048; Similarity, heat effects were higher for fatal MI (RR 1.33, 95% CI 1.24-1.44) than for nonfatal MI (RR 0.99, 95% CI 0.91-1.08), with a *P* value for difference in effect sizes of 0.002. In addition, heat effects were higher for out-of-hospital fatal MI (RR 1.46, 95% CI 1.32-1.61) than for in-hospital fatal MI (RR 1.17, 95% CI 1.05-1.31), with a *P* value for difference in effect sizes of 0.028, whereas the cold effect was not significantly different between out-of-hospital (RR 2.12, 95% CI 1.70-2.64) and in-hospital (RR 1.79, 95% CI 1.40-2.29) fatal MI, with a *P* value for difference in effect sizes of 0.302 (Table 2).

**Table 2:** Cumulative-lag relative risks and 95% confidence intervals (CIs) over 0 to 21 lag days for myocardial infarction (MI) events associated with extreme temperatures

Extreme temperatures	Group	Relative risk∗	Risk difference†		
			d	95% CI	*P* value
Cold effects	Total MI	1.81 (1.59-2.06)			
	Nonfatal MI	1.60 (1.32-1.94)	0.09	(−0.01 to 0.09)	0.048‡
	Fatal MI	1.99 (1.68-2.35)			
	In-hospital fatal MI	1.79 (1.40-2.29)	0.07	(−0.02 to 0.10)	0.302
	Out-of-hospital fatal MI	2.12 (1.70-2.64)			
Heat effects	Total MI	1.17 (1.10-1.24)			
	Nonfatal MI	0.99 (0.91-1.08)	0.13	(0.02-0.09)	0.002‡
	Fatal MI	1.33 (1.24-1.44)			
	In-hospital fatal MI	1.17 (1.05-1.31)	0.10	(0-0.09)	0.028‡
	Out-of-hospital fatal MI	1.46 (1.32-1.61)			

∗ The cold and heat effects were defined as the cumulative-lag risks at the 2.5th percentile (−5.2°C) and the 97.5th percentile (29.6°C) of the temperature distribution relative to the minimum morbidity temperature (24.3°C).

† The difference in relative risks between the 2 groups was calculated as d = log(RR_1_) − log(RR_2_); 95% CIs and *P* values were calculated by 1000-times bootstrap.

‡ *P* < 0.05, suggesting a significant difference in effect sizes.

### MI risk attributable to nonoptimum temperatures

Compared with MMT, the AF of nonoptimum temperatures on total MI was 22.8% (95% CI: 18.3%-26.9%). The AF was higher for fatal MI (25.6%, 95% CI 19.7%-30.6%) than for nonfatal MI (19.1%, 95% CI 12.1%-25.0%) and was higher for out-of-hospital fatal MI (26.8%, 95% CI 18.9%-33.1%) than for in-hospital fatal MI (23.5%, 95% CI 14.5%-31.2%). Moderate cold was responsible for the majority of AF, especially for temperatures from −5.1°C to 5.0°C, with an AF of 11.1% (8.5%-13.5%) for total MI. Similar results were observed in all MI groups (Table 3).

**Table 3:** Attributable fraction (%) and 95% empirical confidence intervals of myocardial infarction events attributable to nonoptimum temperatures∗

Group	Overall	Extreme cold	Moderate cold			Moderate heat	Extreme heat
	−14.3 to 34.5°C	−14.3 to −5.2°C	−5.1 to 5.0°C	5.1 to 15.0°C	15.1 to 24.3°C	24.4 to 29.6°C	29.7 to 34.5°C
Total MI	22.8 (18.3-26.9)	1.9 (1.5-2.2)	11.1 (8.5-13.5)	6.6 (5.0-8.0)	2.7 (1.9-3.4)	0.9 (0.4-1.3)	0.5 (0.4-0.7)
Nonfatal MI	19.1 (12.1-25.0)	1.3 (0.8-1.8)	9.5 (5.7-12.9)	6.2 (3.8-8.4)	2.8 (1.7-3.9)	−0.3 (−1.0 to 0.4)	0 (−0.3 to 0.3)
Fatal MI	25.6 (19.7-30.6)	2.3 (1.8-2.8)	12.4 (9.0-15.5)	6.9 (4.9-8.8)	2.5 (1.6-3.4)	1.7 (1.2-2.3)	0.9 (0.7-1.1)
In-hospital fatal MI	23.5 (14.5-31.2)	1.9 (1.2-2.5)	11.1 (5.7-15.5)	7.1 (4.1-9.9)	3.1 (1.8-4.4)	0.8 (0-1.7)	0.5 (0.2-0.8)
Out-of-hospital fatal MI	26.8 (18.9-33.1)	2.6 (2.0-3.2)	13.2 (8.6-17.2)	6.7 (3.9-9.0)	2.1 (0.9-3.3)	2.3 (1.6-2.9)	1.2 (0.9-1.4)

The 2.5th percentile (−5.2°C), MMT (24.3°C) and 97.5th percentile (29.6°C) were used as cutoff values to classify temperatures into extreme cold (−14.3 to −5.2°C), moderate cold (−5.1 to 24.3°C), moderate heat (24.4 to 29.6°C), and extreme heat (29.7 to 34.5°C). Moderate cold was further divided into 3 sections: −5.1 to 5.0°C, 5.1 to 15.0°C, and 15.1 to 24.3°C.

MI, myocardial infarction.

∗ Monte Carlo simulations were run 5000 times to obtain 95% empirical confidence intervals.

### Subgroup analyses

Similar differences in the associations of temperature with fatal and nonfatal MI were observed in the subgroup analyses. Cold and heat effects were higher for fatal MI than for nonfatal MI, except for cold effects in women. Compared with women, the cumulative cold effects were significantly higher among men for fatal MI (*P* for interaction = 0.018) and in-hospital fatal MI (*P* for interaction = 0.021). In contrast, the cumulative heat effects were significantly higher among women than among men for total MI (*P* for interaction = 0.007), fatal MI (*P* for interaction = 0.008), and out-of-hospital fatal MI (*P* for interaction = 0.013). The cumulative heat effect of total MI was higher in older individuals than in younger individuals (*P* for interaction = 0.023) (Supplemental Fig. S3; Supplemental Table S4). The finer results for age group showed that the heat effects tended to remain stable or slightly elevated with age but peaked with a sudden increase in the ≥ 85 group (Supplemental Table S5).

The AF was higher in men than in women for total MI and fatal MI, including both in-hospital and out-of-hospital fatal MI, but not for nonfatal MI. The AF was higher in older individuals than in younger individuals for total MI, nonfatal MI, and in-hospital fatal MI, but not for fatal MI and out-of-hospital fatal MI (Supplemental Fig. S4; Supplemental Table S6).

### Sensitivity analyses

The models were constructed based on the overdispersion test and Akaike information criterion (Supplemental Tables S7 and S8). The results remained robust under different parameters of the models, different parameters of cold and heat effects, different onset dates, and different block lengths (Supplemental Tables S9-S15). Subgroup analyses showed higher cold effects in patients with NSTEMI than in those with STEMI (Supplemental Table S16). We did not find any significant multiplicative or additive interactions between temperature and PM_2.5_ on fatal and nonfatal MI (Supplemental Table S17).

## Discussion

### Principal findings

In this study, we explored the association between temperature and fatal and nonfatal MI events based on a large sample of citywide registry data. We found that cold and heat were important environmental triggers of MI morbidity. For analyses of the RRs of cold and heat, the single-lag effects had associations that persisted for more days and the cumulative-lag effects were higher for fatal MI than for nonfatal MI. Moreover, population-attributable risks associated with nonoptimum ambient temperatures were higher for fatal MI than for nonfatal MI. Our findings may guide targeted public health policy making to mitigate the hazardous effects of nonoptimum temperatures.

### Comparison with other studies

Significant associations of cold and heat with MI morbidity were found, in agreement with previous studies.^6^, ^7^, ^8^, ^9^ We extended previous findings by demonstrating an apparent difference in the temperature-related risks of fatal and nonfatal MI.^31^ The results from early studies that included both fatal and nonfatal MI were inconsistent, probably owing to relatively small sample sizes (averages of 1 to 3 events per day).^6^, ^7^, ^8^, ^9^ We provided more robust results with multiple sensitivity analyses based on a large population with an average of 87.8 events per day. Another possible reason for the inconsistency of previous findings may be the lack of control for air pollution,^8^^,^^9^ a major confounding factor that may bias the relationship of temperature with fatal and nonfatal MI, with the exceptions of 2 studies that adjusted for PM_10_ in their analyses.^6^^,^^7^ Our study is the first to control for the impact of PM_2.5_, which is considered to be more pathogenic in cardiovascular diseases than PM_10_,^32^ and provides evidence for a difference in the strength of temperature-related association between fatal and nonfatal MI. This may be particularly important for studies on temperature-related health effects in highly polluted cities.

Failure to physiologically adapt to extreme temperatures or sudden temperature changes may explain the more severe deleterious effects of extreme temperature on fatal MI compared with nonfatal MI.^33^ Multiple pathogenetic pathways are triggered directly or indirectly by cold or heat, including hemodynamic effects, inflammation, hydration, sympathetic reactivity, and activation of the renin-angiotensin system.^34^ These rapid biological changes may lead to an acute attack or exacerbation of fatal MI before medical care is obtained or death within hours of exposure, especially when the temperature changes sharply in a short time.^35^ For nonfatal MI, there may be some reasons to explain its weaker association with temperature compared with fatal MI. “Silent MI” is missed within the nonfatal MI cases.^36^ Another explanation could be that nonfatal MI is more likely than fatal MI to occur among more resilient individuals who have lower sensitivity to extreme temperatures. Nevertheless, confirmation of these findings and exploration of the underlying mechanisms require further research.

Another novel finding was that the attributable risk associated with nonoptimum temperatures was higher for fatal MI than for nonfatal MI. Previous studies reported that cardiovascular deaths attributable to temperatures ranged from 10.1% to 23.7%^37^^,^^38^ and that moderate cold was responsible for most of the AF.^26^ However, no previous studies had compared the attributable risk of fatal and nonfatal MI. We found that AF was higher for fatal MI than for nonfatal MI (25.6% vs 19.1%), suggesting that 25.6 in 100 fatal MIs and 19.1 in 100 nonfatal MIs can be attributable to nonoptimum temperatures. Considering the public health implications, especially in a country with a large population such as China, when faced with a population-wide exposure factor such as temperature, it may result in a remarkable difference in the attributable population number of fatal and nonfatal MI attributable to nonoptimum temperatures. In addition, moderate cold temperatures contributed to the largest AF. This can be partly explained by the fact that cold effects were larger and had associations that persisted for more days than heat effects, and moderate cold covered a broad temperature interval, from −5.1°C to 24.3°C, in this study. Notably, temperatures from −5.1°C to 5.0°C showed the largest attributable risk among all temperature categories, especially for fatal MI. This finding supports the current regulation in Beijing that central heating will be turned on when the forecast shows an average temperature of < 5°C for 5 consecutive days.

Temperature-vulnerable populations were identified in this study. Consistently with previous studies, we found that men were more vulnerable to cold and women were more sensitive to heat.^39^ This finding is likely because women tend to have a smaller body size, less body surface area, lower body weight, height, and maximum oxygen consumption, leading to a lower rate of heat exchange and difficulty in heat acclimation.^40^^,^^41^ In contrast, the lower percentage of body fat in men may not be conducive to heat storage in the cold.^42^ In different age groups, we found that older individuals were more vulnerable to heat, which is consistent with a previous meta-analysis.^43^ Impaired thermoregulation and hemodynamic stability with aging may exacerbate heat vulnerability.^44^ Some social factors may also contribute to the vulnerability to heat for the elderly, such as limited access to air conditioning, health care facilities, and social services.^45^

### Policy implications

Our findings may have important implications for health care practitioners, individuals, and governments. First, the development of health warning systems, recognition of early life-threatening symptoms, and rapid accessibility to emergency care could be promoted under extreme temperatures.^46^ Second, vulnerable individuals, such as women and older adults in hot weather and men in cold weather, may be encouraged to take appropriate precautions.^47^ Third, developing relevant policies, such as following strategies to promote safe physical activity in extreme climates^48^ and implementing flexible district heating service policies based on local meteorologic conditions, may protect people from extreme temperatures.^49^

### Limitations

This study has some limitations that must be acknowledged. First, we estimated the health effects only of ambient temperature. Individuals are more likely to stay indoors during extreme outdoor temperatures, and the association between temperature and MI may have been underestimated. Second, this was an ecologic study, and as with previous time-series studies, individual-level confounders were not well controlled, although we controlled for other meteorologic factors, air pollutants, and seasonal and long-term trends. Third, the possibility of diagnosis or coding errors cannot be excluded. However, the diagnostic information has been previously validated with the use of the WHO MONICA criteria based on chart reviews, showing that the data quality is reasonably good.^18^ Finally, although we used data from a citywide registry based on a large sample in Beijing, caution is needed in generalising our findings to other regions.

## Conclusions

The association between temperature and MI morbidity varied between fatal and nonfatal events. The single-lag effects had associations that persisted for more days, the cumulative-lag effects were higher, and the attributable risks were higher for fatal MI than for nonfatal MI. This study may aid targeted policy making to reduce the detrimental effects of nonoptimum temperatures.
