# Supplementary material for: Nonoptimum Temperatures Are More Closely Associated With Fatal Myocardial Infarction Than With Nonfatal Events
Source: Can J Cardiol. 2023 Dec;39(12):1974–83. doi: 10.1016/j.cjca.2023.08.026 (PMC10715678; doi:10.1016/j.cjca.2023.08.026)
Supplement: Supplementary Data [file mmc1.docx]

**SUPPLEMENTARY MATERIAL**

**Nonoptimum Temperatures Are More Closely Associated With Fatal Myocardial**

**Infarction Than With Nonfatal Events**

Piaopiao Hu, MD^a,b,c,d^, Jie Chang, MD^a,b,c,d^, Yulin Huang, MD^a,b,c,d^, Moning Guo, MD^e,f^, Feng Lu, PhD^e,f^, Ying Long, PhD^g^, Huan Liu, PhD^h^, Xudong Yang, PhD^i^, Yue Qi, MD, PhD^a,b,c,d^, Jiayi Sun, MD^a,b,c,d^, Zhao Yang, PhD^a,b,c,d^, Qiuju Deng, MD, PhD^a,b,c,d,^*, qiujudeng@mail.ccmu.edu.cn, Jing Liu, MD, PhD^a,b,c,d,^*, jingliu@ccmu.edu.cn

^a^Center for Clinical and Epidemiologic Research, Beijing An Zhen Hospital, Capital Medical University, Beijing Institute of Heart, Lung, and Blood Vessel Diseases, Beijing, China

^b^National Clinical Research Center of Cardiovascular Diseases, Beijing, China

^c^Key Laboratory of Remodeling-Related Cardiovascular Diseases, Ministry of Education, Beijing, China

^d^Beijing Municipal Key Laboratory of Clinical Epidemiology, Beijing, China

^e^Beijing Municipal Health Big Data and Policy Research Center, Beijing, China

^f^Beijing Institute of Hospital Management, Beijing, China

^g^School of Architecture, Tsinghua University, Beijing, China

^h^State Key Joint Laboratory of ESPC, State Environmental Protection Key Laboratory of Sources and Control of Air Pollution Complex, School of Environment, Tsinghua University, Beijing, China

^i^Departments of Building Science and Vanke School of Public Health, Tsinghua University, Beijing, China

∗Corresponding author: Dr Jing Liu, Beijing Institute of Heart, Lung, and Blood Vessel Diseases, Beijing An Zhen Hospital, Capital Medical University, No. 2 An Zhen Road, Chaoyang District, Beijing 100029, China. Tel.: +86 10-64456710, fax: +86 10-64456710.

∗Corresponding author: Dr Qiuju Deng, Beijing Institute of Heart, Lung, and Blood Vessel Diseases, Beijing An Zhen Hospital, Capital Medical University, No. 2 An Zhen Road, Chaoyang District, Beijing 100029, China. Tel.: +86 10-64456324, fax: +86 10-64456710.

**Contents**

**Supplemental Table S1.** Descriptive statistics of the study population and myocardial infarction counts.

**Supplemental Table S2.** Overall lag structure in relative risks of extreme cold temperatures on myocardial infarction events.

**Supplemental Table S3.** Overall lag structure in relative risks of extreme heat temperatures on myocardial infarction events.

**Supplemental Table S4.** Cumulative-lag relative risks and 95% confidence intervals over 0 to 21 lag days for myocardial infarction events associated with extreme temperatures in sex and age groups (<65 and ≥65 years).

**Supplemental Table S5.** Cumulative-lag relative risks and 95% confidence intervals over 0 to 21 lag days for myocardial infarction events associated with extreme temperatures in age groups (**<**65, 65–74, 75–84 and ≥85 years).

**Supplemental Table S6.** Attributable fraction (%) and 95% empirical confidence intervals of myocardial infarction events attributable to temperatures in sex and age groups.

**Supplemental Table S7.** Overdispersion tests for fitting different models of the association between myocardial infarction events and extreme temperatures.

**Supplemental Table S8.** Akaike information criterion value of different choices of df for temperature space and lag space in the distributed-lag nonlinear model.

**Supplemental Table S9.** Cumulative-lag relative risks and 95% confidence intervals over 0 to 21 lag days for myocardial infarction events associated with extreme temperatures by changing the degrees of freedom for time (6, 8, and 10) per year.

**Supplemental Table S10.** Cumulative-lag relative risks and 95% confidence intervals over 0 to 21 lag days for myocardial infarction events associated with extreme temperatures in the unadjusted and confounder-adjusted models.

**Supplemental Table S11.** Cumulative-lag relative risks and 95% confidence intervals for myocardial infarction events associated with extreme temperatures by changing the maximum lag period (4, 14, 28, and 31 days).

**Supplemental Table S12.** Cumulative-lag relative risks and 95% confidence intervals over 0 to 21 lag days for myocardial infarction events associated with extreme temperatures using different temperature cutoffs (first/99th percentiles, 5th/95th percentiles and 10th/90th percentiles).

**Supplemental Table S13.** Cumulative-lag relative risks and 95% confidence intervals over 0 to 21 lag days for myocardial infarction events associated with extreme temperatures using different reference values (25th and 75th percentiles).

**Supplemental Table S14.** Cumulative-lag relative risks and 95% confidence intervals over 0 to 21 lag days for myocardial infarction events associated with extreme temperatures using the onset time of the last event within 28 days.

**Supplemental Table S15.** Cumulative-lag relative risks and 95% confidence intervals over 0 to 21 lag days for myocardial infarction events associated with extreme temperatures changing the block length in the stationary block bootstrap method.

**Supplemental Table S16.** Cumulative-lag relative risks and 95% confidence intervals over 0 to 21 lag days for myocardial infarction events associated with extreme temperatures stratified by subgroup (types of MI, payment methods, comorbidities, and reperfusion therapy).

**Supplemental Table S17.** Interactive and stratified analyses of temperature and PM_2.5_ on myocardial infarction events.

**Supplemental Figure S1.** Flow diagram of myocardial infarction events enrolled in the Beijing Cardiovascular Disease Surveillance System.

**Supplemental Figure S2.** Time series plots of daily myocardial infarction events, meteorological factors, and PM_2.5_ concentrations in Beijing, China, 2007−2019.

**Supplemental Figure S3.** Cumulative-lag relative risks and 95% confidence intervals of cold and heat effects on myocardial infarction events over 0 to 21 lag days in sex and age groups.

**Supplemental Figure S4.** Attributable fraction (%) of myocardial infarction events attributable to nonoptimum temperatures in sex and age groups.

**Supplemental Tables**

**Supplemental Table S1. Descriptive statistics of the study population and myocardial infarction counts.**

| Variables | Value |
| --- | --- |
| Age, mean (SD), year | 72.2 (13.5) |
| Age group, n (%) |  |
| <65 years | 119,987 (28.8) |
| ≥65 years | 296,907 (71.2) |
| Sex, n (%) |  |
| Men | 250,595 (60.1) |
| Women | 166,299 (39.9) |
| Total MI counts, n (%) | 416,894 (100.0) |
| Nonfatal MI | 183,823 (44.1) |
| Fatal MI | 233,071 (55.9) |
| In-hospital fatal MI | 95,043 (22.8) |
| Out-of-hospital fatal MI | 138,028 (33.1) |

MI, myocardial infarction; SD, standard deviation.

**Supplemental Table S2. Overall lag structure in relative risks of extreme cold temperatures on myocardial infarction events. ^a^**

| Lag (day) | Total MI | Nonfatal MI | Fatal MI | In-hospital fatal MI | Out-of-hospital fatal MI |
| --- | --- | --- | --- | --- | --- |
| 0 | 0.85 (0.81 to 0.89) | 0.79 (0.73 to 0.85) | 0.90 (0.84 to 0.96) | 0.86 (0.78 to 0.95) | 0.93 (0.85 to 1.01) |
| 1 | 1.03 (1.00 to 1.05) | 1.04 (1.01 to 1.08) | 1.01 (0.98 to 1.04) | 1.01 (0.96 to 1.05) | 1.02 (0.98 to 1.06) |
| 2 | 1.09 (1.07 to 1.12) | 1.13 (1.09 to 1.17) | 1.07 (1.03 to 1.10) | 1.07 (1.01 to 1.12) | 1.07 (1.02 to 1.12) |
| 3 | 1.08 (1.07 to 1.10) | 1.10 (1.07 to 1.12) | 1.08 (1.06 to 1.10) | 1.06 (1.04 to 1.09) | 1.08 (1.06 to 1.11) |
| 4 | 1.07 (1.06 to 1.09) | 1.07 (1.05 to 1.09) | 1.08 (1.06 to 1.09) | 1.06 (1.03 to 1.08) | 1.09 (1.07 to 1.11) |
| 5 | 1.06 (1.05 to 1.08) | 1.05 (1.03 to 1.07) | 1.07 (1.05 to 1.09) | 1.05 (1.03 to 1.08) | 1.09 (1.06 to 1.11) |
| 6 | 1.06 (1.04 to 1.07) | 1.05 (1.03 to 1.07) | 1.07 (1.05 to 1.08) | 1.05 (1.02 to 1.07) | 1.08 (1.06 to 1.10) |
| 7 | 1.05 (1.04 to 1.06) | 1.04 (1.03 to 1.06) | 1.06 (1.05 to 1.07) | 1.05 (1.02 to 1.07) | 1.07 (1.05 to 1.09) |
| 8 | 1.05 (1.04 to 1.06) | 1.04 (1.03 to 1.06) | 1.05 (1.04 to 1.06) | 1.04 (1.03 to 1.06) | 1.06 (1.04 to 1.07) |
| 9 | 1.05 (1.04 to 1.05) | 1.04 (1.03 to 1.06) | 1.05 (1.04 to 1.06) | 1.04 (1.02 to 1.06) | 1.05 (1.04 to 1.07) |
| 10 | 1.04 (1.03 to 1.05) | 1.04 (1.03 to 1.05) | 1.04 (1.03 to 1.05) | 1.04 (1.02 to 1.05) | 1.04 (1.03 to 1.06) |
| 11 | 1.04 (1.03 to 1.05) | 1.04 (1.03 to 1.05) | 1.04 (1.02 to 1.05) | 1.03 (1.02 to 1.05) | 1.04 (1.02 to 1.05) |
| 12 | 1.03 (1.02 to 1.04) | 1.04 (1.02 to 1.05) | 1.03 (1.02 to 1.04) | 1.03 (1.01 to 1.05) | 1.03 (1.02 to 1.05) |
| 13 | 1.03 (1.02 to 1.04) | 1.03 (1.02 to 1.04) | 1.03 (1.02 to 1.04) | 1.03 (1.01 to 1.05) | 1.03 (1.01 to 1.04) |
| 14 | 1.03 (1.02 to 1.03) | 1.03 (1.01 to 1.04) | 1.03 (1.01 to 1.04) | 1.03 (1.01 to 1.05) | 1.02 (1.01 to 1.04) |
| 15 | 1.02 (1.01 to 1.03) | 1.02 (1.01 to 1.03) | 1.02 (1.01 to 1.03) | 1.03 (1.01 to 1.04) | 1.02 (1.01 to 1.03) |
| 16 | 1.02 (1.01 to 1.03) | 1.01 (1.00 to 1.03) | 1.02 (1.01 to 1.03) | 1.03 (1.01 to 1.04) | 1.02 (1.00 to 1.03) |
| 17 | 1.01 (1.01 to 1.02) | 1.01 (1.00 to 1.02) | 1.02 (1.01 to 1.03) | 1.02 (1.01 to 1.04) | 1.01 (1.00 to 1.03) |
| 18 | 1.01 (1.00 to 1.02) | 1.00 (0.99 to 1.01) | 1.02 (1.00 to 1.03) | 1.02 (1.01 to 1.04) | 1.01 (1.00 to 1.03) |
| 19 | 1.01 (0.99 to 1.02) | 0.99 (0.98 to 1.01) | 1.02 (1.00 to 1.03) | 1.02 (1.00 to 1.04) | 1.01 (0.99 to 1.03) |
| 20 | 1.00 (0.99 to 1.02) | 0.99 (0.97 to 1.01) | 1.01 (1.00 to 1.03) | 1.02 (0.99 to 1.05) | 1.01 (0.99 to 1.03) |
| 21 | 1.00 (0.98 to 1.01) | 0.98 (0.96 to 1.00) | 1.01 (0.99 to 1.03) | 1.02 (0.99 to 1.05) | 1.01 (0.98 to 1.03) |

MI, myocardial infarction.

^a^ The relative risks were defined as the single-lag effects at the 2.5th percentile (−5.2°C) of the temperature distribution relative to the minimum morbidity temperature (24.3°C).

**Supplemental Table S3. Overall lag structure in relative risks of extreme heat temperatures on myocardial infarction events. ^a^**

| Lag (day) | Total MI | Nonfatal MI | Fatal MI | In-hospital fatal MI | Out-of-hospital fatal MI |
| --- | --- | --- | --- | --- | --- |
| 0 | 1.05 (1.03 to 1.07) | 1.04 (1.01 to 1.07) | 1.05 (1.03 to 1.08) | 1.03 (0.99 to 1.07) | 1.07 (1.03 to 1.11) |
| 1 | 1.04 (1.03 to 1.05) | 1.01 (0.99 to 1.02) | 1.06 (1.05 to 1.08) | 1.03 (1.01 to 1.05) | 1.09 (1.07 to 1.11) |
| 2 | 1.03 (1.02 to 1.04) | 1.00 (0.98 to 1.01) | 1.05 (1.04 to 1.07) | 1.03 (1.01 to 1.05) | 1.07 (1.05 to 1.09) |
| 3 | 1.02 (1.01 to 1.02) | 0.99 (0.98 to 1.00) | 1.03 (1.03 to 1.04) | 1.02 (1.01 to 1.03) | 1.04 (1.03 to 1.05) |
| 4 | 1.01 (1.00 to 1.01) | 0.99 (0.99 to 1.00) | 1.02 (1.01 to 1.03) | 1.02 (1.00 to 1.03) | 1.03 (1.02 to 1.03) |
| 5 | 1.00 (1.00 to 1.01) | 0.99 (0.99 to 1.00) | 1.01 (1.01 to 1.02) | 1.01 (1.00 to 1.02) | 1.01 (1.00 to 1.02) |
| 6 | 1.00 (1.00 to 1.01) | 0.99 (0.99 to 1.00) | 1.01 (1.00 to 1.02) | 1.01 (1.00 to 1.02) | 1.01 (1.00 to 1.02) |
| 7 | 1.00 (1.00 to 1.01) | 1.00 (0.99 to 1.00) | 1.01 (1.00 to 1.01) | 1.01 (1.00 to 1.01) | 1.01 (1.00 to 1.02) |
| 8 | 1.00 (1.00 to 1.01) | 1.00 (0.99 to 1.00) | 1.01 (1.00 to 1.01) | 1.00 (1.00 to 1.01) | 1.01 (1.00 to 1.01) |
| 9 | 1.00 (1.00 to 1.01) | 1.00 (0.99 to 1.00) | 1.01 (1.00 to 1.01) | 1.00 (1.00 to 1.01) | 1.01 (1.00 to 1.01) |
| 10 | 1.00 (1.00 to 1.01) | 1.00 (0.99 to 1.00) | 1.00 (1.00 to 1.01) | 1.00 (0.99 to 1.01) | 1.01 (1.00 to 1.01) |
| 11 | 1.00 (1.00 to 1.01) | 1.00 (0.99 to 1.00) | 1.00 (1.00 to 1.01) | 1.00 (0.99 to 1.01) | 1.01 (1.00 to 1.01) |
| 12 | 1.00 (1.00 to 1.01) | 1.00 (0.99 to 1.01) | 1.00 (1.00 to 1.01) | 1.00 (0.99 to 1.01) | 1.01 (1.00 to 1.01) |
| 13 | 1.00 (1.00 to 1.01) | 1.00 (0.99 to 1.01) | 1.00 (1.00 to 1.01) | 1.00 (0.99 to 1.01) | 1.01 (1.00 to 1.01) |
| 14 | 1.00 (1.00 to 1.01) | 1.00 (0.99 to 1.00) | 1.00 (1.00 to 1.01) | 1.00 (0.99 to 1.01) | 1.00 (1.00 to 1.01) |
| 15 | 1.00 (1.00 to 1.00) | 1.00 (0.99 to 1.00) | 1.00 (1.00 to 1.01) | 1.00 (0.99 to 1.01) | 1.00 (1.00 to 1.01) |
| 16 | 1.00 (1.00 to 1.00) | 1.00 (0.99 to 1.00) | 1.00 (1.00 to 1.01) | 1.00 (0.99 to 1.01) | 1.00 (1.00 to 1.01) |
| 17 | 1.00 (1.00 to 1.00) | 1.00 (0.99 to 1.00) | 1.00 (1.00 to 1.01) | 1.00 (0.99 to 1.01) | 1.00 (1.00 to 1.01) |
| 18 | 1.00 (1.00 to 1.00) | 1.00 (0.99 to 1.00) | 1.00 (1.00 to 1.01) | 1.00 (0.99 to 1.01) | 1.00 (0.99 to 1.01) |
| 19 | 1.00 (1.00 to 1.00) | 1.00 (0.99 to 1.00) | 1.00 (0.99 to 1.01) | 1.00 (0.99 to 1.01) | 1.00 (0.99 to 1.01) |
| 20 | 1.00 (0.99 to 1.00) | 1.00 (0.99 to 1.01) | 1.00 (0.99 to 1.01) | 1.00 (0.99 to 1.01) | 1.00 (0.99 to 1.01) |
| 21 | 1.00 (0.99 to 1.01) | 1.00 (0.99 to 1.01) | 1.00 (0.99 to 1.01) | 1.00 (0.99 to 1.02) | 1.00 (0.99 to 1.01) |

MI, myocardial infarction.

^a^ The relative risks were defined as the single-lag effects at the 97.5th percentile (29.6°C) of the temperature distribution relative to the minimum morbidity temperature (24.3°C). **Supplemental Table S4. Cumulative-lag relative risks and 95% confidence intervals over 0 to 21 lag days for myocardial infarction events associated with extreme temperatures in sex and age groups (<65 and ≥65 years).**

|  | Group | Sex | | | Age | | |
| --- | --- | --- | --- | --- | --- | --- | --- |
|  |  | Men | Women | *P* value | <65 years | ≥65 years | *P* value |
| Cold effects ^a^ | Total MI | 1.94 (1.65 to 2.27) | 1.63 (1.34 to 1.99) | 0.179 | 1.58 (1.26 to 1.98) | 1.91 (1.64 to 2.23) | 0.174 |
|  | Nonfatal MI | 1.57 (1.26 to 1.96) | 1.65 (1.19 to 2.29) | 0.805 | 1.32 (1.02 to 1.72) | 1.88 (1.46 to 2.44) | 0.058 |
|  | Fatal MI | 2.39 (1.91 to 2.98) | 1.61 (1.27 to 2.05) | 0.018* | 2.48 (1.63 to 3.77) | 1.91 (1.60 to 2.30) | 0.263 |
|  | In-hospital fatal MI | 2.33 (1.67 to 3.25) | 1.30 (0.90 to 1.88) | 0.021* | 1.61 (0.82 to 3.18) | 1.81 (1.39 to 2.37) | 0.753 |
|  | Out-of-hospital fatal MI | 2.41 (1.79 to 3.23) | 1.85 (1.36 to 2.51) | 0.223 | 3.16 (1.87 to 5.35) | 1.97 (1.55 to 2.50) | 0.109 |
| Heat effects ^a^ | Total MI | 1.10 (1.02 to 1.18) | 1.29 (1.18 to 1.41) | 0.007* | 1.06 (0.96 to 1.17) | 1.22 (1.14 to 1.31) | 0.023* |
|  | Nonfatal MI | 1.00 (0.91 to 1.10) | 0.98 (0.85 to 1.14) | 0.821 | 0.99 (0.88 to 1.11) | 0.99 (0.88 to 1.12) | 1.000 |
|  | Fatal MI | 1.21 (1.09 to 1.34) | 1.48 (1.33 to 1.65) | 0.008* | 1.26 (1.04 to 1.52) | 1.35 (1.24 to 1.46) | 0.513 |
|  | In-hospital fatal MI | 1.10 (0.94 to 1.28) | 1.26 (1.07 to 1.49) | 0.240 | 1.13 (0.83 to 1.54) | 1.18 (1.05 to 1.33) | 0.798 |
|  | Out-of-hospital fatal MI | 1.30 (1.14 to 1.48) | 1.65 (1.44 to 1.89) | 0.013* | 1.35 (1.06 to 1.70) | 1.48 (1.33 to 1.64) | 0.486 |

MI, myocardial infarction; df, degrees of freedom.

^a^ The cold and heat effects were defined as the cumulative-lag risks at the 2.5th percentile (−5.2°C) and the 97.5th percentile (29.6°C) of the temperature distribution relative to the minimum morbidity temperature (24.3°C), respectively. Asterisks indicate *P*<0.05, suggesting a significant interaction.

**Supplemental Table S5. Cumulative-lag relative risks and 95% confidence intervals over 0 to 21 lag days for myocardial infarction events associated with extreme temperatures in age groups** **(<65, 65**–**74, 75**–**84 and ≥85 years).**

|  | Group | Age group, years | | | |
| --- | --- | --- | --- | --- | --- |
|  |  | <65 | 65-74 | 75-84 | ≥85 |
| Cold effects ^a^ | Total MI | 1.58 (1.26 to 1.98) | 1.80 (1.38 to 2.35) | 2.13 (1.71 to 2.65) | 1.71 (1.30 to 2.26) |
|  | Nonfatal MI | 1.32 (1.02 to 1.72) | 1.92 (1.33 to 2.78) | 2.08 (1.43 to 3.02) | 1.14 (0.55 to 2.34) |
|  | Fatal MI | 2.48 (1.63 to 3.77) | 1.63 (1.12 to 2.37) | 2.13 (1.63 to 2.77) | 1.84 (1.37 to 2.48) |
|  | In-hospital fatal MI | 1.61 (0.82 to 3.18) | 1.73 (0.97 to 3.09) | 1.75 (1.19 to 2.57) | 1.98 (1.24 to 3.16) |
|  | Out-of-hospital fatal MI | 3.16 (1.87 to 5.35) | 1.54 (0.94 to 2.54) | 2.43 (1.72 to 3.45) | 1.75 (1.20 to 2.55) |
| Heat effects ^a^ | Total MI | 1.06 (0.96 to 1.17) | 1.10 (0.97 to 1.24) | 1.17 (1.06 to 1.30) | 1.45 (1.28 to 1.65)* |
|  | Nonfatal MI | 0.99 (0.88 to 1.11) | 1.00 (0.85 to 1.18) | 0.95 (0.80 to 1.13) | 1.17 (0.84 to 1.62) |
|  | Fatal MI | 1.26 (1.04 to 1.52) | 1.21 (1.02 to 1.44) | 1.29 (1.15 to 1.46) | 1.51 (1.32 to 1.72) |
|  | In-hospital fatal MI | 1.13 (0.83 to 1.54) | 1.01 (0.78 to 1.32) | 1.26 (1.06 to 1.50) | 1.18 (0.96 to 1.45) |
|  | Out-of-hospital fatal MI | 1.35 (1.06 to 1.70) | 1.38 (1.11 to 1.73) | 1.32 (1.13 to 1.55) | 1.76 (1.48 to 2.09) |

MI, myocardial infarction.

^a^ The cold and heat effects were defined as the cumulative-lag risks at the 2.5th percentile (−5.2°C) and the 97.5th percentile (29.6°C) of the temperature distribution relative to the minimum morbidity temperature (24.3°C), respectively. The <65 years group was used as the reference. Asterisks indicate *P*<0.05, suggesting a significant interaction.

**Supplemental Table S6. Attributable fraction (%) and 95% empirical confidence intervals of myocardial infarction events attributable to temperatures in sex and age groups. ^a^**

| Group | Temperature range (°C) | Sex | | Age groups | |
| --- | --- | --- | --- | --- | --- |
|  |  | Men | Women | <65 years | ≥65 years |
| Total MI | −14.3 to 34.5 | 24.6 (19.5 to 29.4) | 19.7 (11.6 to 26.4) | 15.8 (7.0 to 23.2) | 25.5 (20.1 to 30.2) |
|  | −14.3 to −5.2 | 2.0 (1.5 to 2.4) | 1.7 (1.1 to 2.2) | 1.4 (0.7 to 1.9) | 2.1 (1.7 to 2.5) |
|  | −5.1 to 5.0 | 12.4 (9.3 to 15.0) | 8.9 (4.4 to 12.7) | 8.5 (3.6 to 12.7) | 12.2 (9.1 to 14.8) |
|  | 5.1 to 15.0 | 7.4 (5.4 to 9.2) | 5.3 (2.7 to 7.6) | 4.2 (1.1 to 6.9) | 7.5 (5.7 to 9.2) |
|  | 15.1 to 24.3 | 3.1 (2.3 to 4.0) | 1.9 (0.8 to 3.0) | 1.6 (0.3 to 2.8) | 3.1 (2.3 to 3.9) |
|  | 24.4 to 29.6 | 0.4 (−0.2 to 0.9) | 1.6 (0.9 to 2.2) | 0.3 (−0.5 to 1.1) | 1.1 (0.6 to 1.6) |
|  | 29.7 to 34.5 | 0.3 (0.1 to 0.6) | 0.8 (0.6 to 1.1) | 0.2 (−0.1 to 0.5) | 0.6 (0.5 to 0.8) |
| Nonfatal MI | −14.3 to 34.5 | 18.5 (10.3 to 25.3) | 20.3 (8.0 to 30.6) | 10.1 (−1.2 to 19.4) | 26.1 (17.6 to 32.8) |
|  | −14.3 to −5.2 | 1.2 (0.6 to 1.8) | 1.5 (0.5 to 2.3) | 0.8 (0 to 1.4) | 1.8 (1.1 to 2.4) |
|  | −5.1 to 5.0 | 9.3 (4.8 to 13.2) | 9.8 (2.6 to 15.3) | 5.8 (−0.5 to 10.9) | 12.4 (7.5 to 16.3) |
|  | 5.1 to 15.0 | 6.0 (3.2 to 8.5) | 6.6 (2.5 to 10.2) | 2.7 (−0.9 to 6.0) | 9.0 (6.1 to 11.7) |
|  | 15.1 to 24.3 | 2.6 (1.4 to 3.8) | 3.3 (1.4 to 5.0) | 1.0 (−0.7 to 2.5) | 4.4 (3.0 to 5.8) |
|  | 24.4 to 29.6 | −0.2 (−1.0 to 0.6) | −0.4 (−1.6 to 0.7) | −0.1 (−1.2 to 0.8) | −0.4 (−1.3 to 0.5) |
|  | 29.7 to 34.5 | 0 (−0.3 to 0.3) | 0 (−0.4 to 0.4) | 0 (−0.4 to 0.4) | 0 (−0.3 to 0.4) |
| Fatal MI | −14.3 to 34.5 | 30.5 (23.3 to 36.3) | 18.9 (8.9 to 27.6) | 29.2 (14.6 to 39.6) | 24.9 (18.2 to 30.5) |
|  | −14.3 to −5.2 | 2.8 (2.2 to 3.3) | 1.7 (1.0 to 2.4) | 3.0 (1.8 to 3.9) | 2.2 (1.7 to 2.7) |
|  | −5.1 to 5.0 | 15.6 (11.5 to 19.1) | 8.2 (2.4 to 13.2) | 15.2 (6.8 to 21.2) | 11.9 (8.1 to 15.3) |
|  | 5.1 to 15.0 | 8.9 (6.4 to 11.2) | 4.5 (1.2 to 7.3) | 7.9 (2.7 to 12.0) | 6.7 (4.4 to 8.8) |
|  | 15.1 to 24.3 | 3.6 (2.4 to 4.7) | 1.2 (−0.1 to 2.5) | 3.1 (0.7 to 5.2) | 2.4 (1.4 to 3.4) |
|  | 24.4 to 29.6 | 1.0 (0.3 to 1.7) | 2.5 (1.8 to 3.2) | 1.4 (−0.1 to 2.7) | 1.8 (1.2 to 2.4) |
|  | 29.7 to 34.5 | 0.6 (0.3 to 0.9) | 1.2 (0.9 to 1.5) | 0.7 (0.2 to 1.2) | 0.9 (0.7 to 1.2) |
| In-hospital fatal MI | −14.3 to 34.5 | 29.0 (18.5 to 37.5) | 15.5 (−1.3 to 28.6) | 18.0 (−14.5 to 37.5) | 24.3 (14.6 to 32.1) |
|  | −14.3 to −5.2 | 2.6 (1.7 to 3.4) | 0.9 (−0.2 to 1.9) | 1.6 (−0.6 to 3.2) | 1.9 (1.2 to 2.6) |
|  | −5.1 to 5.0 | 14.9 (8.8 to 19.8) | 5.4 (−4.4 to 13.2) | 7.9 (−11.4 to 19.3) | 11.5 (5.6 to 16.2) |
|  | 5.1 to 15.0 | 8.8 (4.6 to 12.2) | 5.1 (0 to 9.4) | 5.3 (−5.2 to 12.9) | 7.4 (4.0 to 10.3) |
|  | 15.1 to 24.3 | 3.8 (2.0 to 5.5) | 2.2 (0.1 to 4.1) | 2.5 (−1.4 to 6.0) | 3.2 (1.7 to 4.6) |
|  | 24.4 to 29.6 | 0.3 (−0.8 to 1.4) | 1.4 (0.2 to 2.6) | 0.7 (−1.8 to 2.8) | 0.9 (0 to 1.7) |
|  | 29.7 to 34.5 | 0.3 (−0.1 to 0.8) | 0.7 (0.3 to 1.2) | 0.4 (−0.5 to 1.3) | 0.5 (0.2 to 0.9) |
| Out-of-hospital fatal MI | −14.3 to 34.5 | 31.3 (21.9 to 39.1) | 21.1 (8.3 to 31.3) | 34.6 (18.3 to 45.5) | 25.0 (16.1 to 32.4) |
|  | −14.3 to −5.2 | 2.9 (2.1 to 3.7) | 2.3 (1.3 to 3.1) | 3.7 (2.3 to 4.9) | 2.4 (1.7 to 3.1) |
|  | −5.1 to 5.0 | 15.9 (10.2 to 20.5) | 9.9 (2.6 to 15.8) | 18.7 (9.6 to 25.1) | 12.0 (6.8 to 16.4) |
|  | 5.1 to 15.0 | 8.9 (5.4 to 11.9) | 4.1 (0 to 7.7) | 9.1 (3.1 to 13.9) | 6.2 (3.3 to 8.8) |
|  | 15.1 to 24.3 | 3.4 (1.9 to 4.9) | 0.6 (−1.1 to 2.3) | 3.4 (0.4 to 6.1) | 1.9 (0.5 to 3.2) |
|  | 24.4 to 29.6 | 1.4 (0.5 to 2.3) | 3.2 (2.3 to 4.0) | 1.8 (0 to 3.4) | 2.4 (1.7 to 3.1) |
|  | 29.7 to 34.5 | 0.8 (0.5 to 1.2) | 1.5 (1.2 to 1.9) | 0.9 (0.3 to 1.5) | 1.2 (0.9 to 1.5) |

MI, myocardial infarction.

^a^ Empirical confidence intervals were calculated by Monte Carlo simulations (5000 random samples).

**Supplemental Table S7. Overdispersion tests for fitting different models of the association between myocardial infarction events and extreme temperatures.**

| Model | deviance | df.residual | deviance/ df.residual | *P* value ^a^ |
| --- | --- | --- | --- | --- |
| Poisson regression | 5709.6 | 4603 | 1.24 | <0.001 |
| Quasi-Poisson regression | 5709.6 | 4603 | 1.24 | <0.001 |
| Negative Binomial regression | 4621.0 | 4603 | 1.00 | 0.423 |

MI, myocardial infarction; df, degrees of freedom.

^a^ *P*<0.05 indicates overdispersion.

**Supplemental Table S8. Akaike information criterion value of different choices of df for temperature space and lag space in the distributed-lag nonlinear model.**

| df for temperature | df for lag | AIC |
| --- | --- | --- |
| 3 | 3 | 35738 |
| 3 | 4 | 35724 |
| 3 | 5 | 35722 |
| 3 | 6 | 35720 |
| 4 | 3 | 35562 |
| 4 | 4 | 35555* |
| 4 | 5 | 35557 |
| 4 | 6 | 35556 |
| 5 | 3 | 35565 |
| 5 | 4 | 35559 |
| 5 | 5 | 35558 |
| 5 | 6 | 35559 |
| 6 | 3 | 35564 |
| 6 | 4 | 35560 |
| 6 | 5 | 35562 |
| 6 | 6 | 35564 |

df, degrees of freedom; AIC, Akaike information criterion.

Asterisk indicates the model constraints with the lowest AlC.

**Supplemental Table S9. Cumulative-lag relative risks and 95% confidence intervals over 0 to 21 lag days for myocardial infarction events associated with extreme temperatures by changing the degrees of freedom for time (6, 8, and 10) per year.**

| df for time | Group | Cold effects ^a^ | Heat effects ^a^ |
| --- | --- | --- | --- |
| df=6 | Total MI | 1.78 (1.59 to 1.99) | 1.10 (1.05 to 1.16) |
|  | Nonfatal MI | 1.75 (1.48 to 2.06) | 0.98 (0.91 to 1.05) |
|  | Fatal MI | 1.79 (1.54 to 2.07) | 1.21 (1.14 to 1.29) |
|  | In-hospital fatal MI | 1.74 (1.40 to 2.15) | 1.12 (1.02 to 1.23) |
|  | Out-of-hospital fatal MI | 1.80 (1.49 to 2.18) | 1.27 (1.17 to 1.38) |
| df=8 | Total MI | 1.74 (1.52 to 1.99) | 1.16 (1.09 to 1.24) |
|  | Nonfatal MI | 1.55 (1.28 to 1.89) | 0.98 (0.89 to 1.08) |
|  | Fatal MI | 1.88 (1.58 to 2.24) | 1.34 (1.23 to 1.46) |
|  | In-hospital fatal MI | 1.77 (1.37 to 2.29) | 1.18 (1.04 to 1.34) |
|  | Out-of-hospital fatal MI | 1.94 (1.54 to 2.44) | 1.45 (1.30 to 1.62) |
| df=10 | Total MI | 1.61 (1.38 to 1.88) | 1.17 (1.10 to 1.25) |
|  | Nonfatal MI | 1.58 (1.26 to 1.98) | 0.99 (0.90 to 1.09) |
|  | Fatal MI | 1.64 (1.35 to 2.01) | 1.35 (1.23 to 1.47) |
|  | In-hospital fatal MI | 1.38 (1.03 to 1.86) | 1.14 (1.00 to 1.30) |
|  | Out-of-hospital fatal MI | 1.85 (1.43 to 2.40) | 1.51 (1.35 to 1.69) |

MI, myocardial infarction; df, degrees of freedom.

^a^ The cold and heat effects were defined as the cumulative-lag risks at the 2.5th percentile (−5.2°C) and the 97.5th percentile (29.6°C) of the temperature distribution relative to the minimum morbidity temperature (24.3°C), respectively.

**Supplemental Table S10. Cumulative-lag relative risks and 95% confidence intervals over 0 to 21 lag days for myocardial infarction events associated with extreme temperatures in the unadjusted and confounder-adjusted models.**

|  | Group | Cold effects ^a^ | Heat effects ^a^ |
| --- | --- | --- | --- |
| Model 1 ^b^ | Total MI | 1.51 (1.46 to 1.56) | 1.21 (1.16 to 1.26) |
|  | Nonfatal MI | 1.29 (1.23 to 1.35) | 1.17 (1.10 to 1.23) |
|  | Fatal MI | 1.69 (1.64 to 1.75) | 1.24 (1.19 to 1.29) |
|  | In-hospital fatal MI | 1.55 (1.48 to 1.61) | 1.23 (1.16 to 1.30) |
|  | Out-of-hospital fatal MI | 1.80 (1.74 to 1.86) | 1.24 (1.19 to 1.30) |
| Model 2 ^b^ | Total MI | 1.79 (1.57 to 2.03) | 1.17 (1.10 to 1.24) |
|  | Nonfatal MI | 1.61 (1.33 to 1.95) | 0.99 (0.91 to 1.07) |
|  | Fatal MI | 1.92 (1.63 to 2.27) | 1.34 (1.25 to 1.45) |
|  | In-hospital fatal MI | 1.70 (1.33 to 2.17) | 1.19 (1.07 to 1.33) |
|  | Out-of-hospital fatal MI | 2.08 (1.67 to 2.59) | 1.46 (1.32 to 1.61) |
| Model 3 ^b^ | Total MI | 1.81 (1.59 to 2.06) | 1.17 (1.10 to 1.24) |
|  | Nonfatal MI | 1.60 (1.32 to 1.94) | 0.99 (0.91 to 1.08) |
|  | Fatal MI | 1.99 (1.68 to 2.35) | 1.33 (1.24 to 1.44) |
|  | In-hospital fatal MI | 1.79 (1.40 to 2.29) | 1.17 (1.05 to 1.31) |
|  | Out-of-hospital fatal MI | 2.12 (1.70 to 2.64) | 1.46 (1.32 to 1.61) |
| Model 4 ^b^ | Total MI | 2.65 (1.84 to 3.82) | 1.19 (1.04 to 1.35) |
|  | Nonfatal MI | 2.20 (1.28 to 3.78) | 0.92 (0.76 to 1.11) |
|  | Fatal MI | 3.04 (1.90 to 4.87) | 1.45 (1.23 to 1.72) |
|  | In-hospital fatal MI | 2.60 (1.29 to 5.21) | 1.29 (1.01 to 1.66) |
|  | Out-of-hospital fatal MI | 3.33 (1.81 to 6.14) | 1.57 (1.26 to 1.96) |
| Model 5 ^b^ | Total MI | 2.80 (1.94 to 4.05) | 1.16 (1.02 to 1.32) |
|  | Nonfatal MI | 2.32 (1.35 to 3.99) | 0.90 (0.75 to 1.09) |
|  | Fatal MI | 3.24 (2.02 to 5.20) | 1.42 (1.20 to 1.67) |
|  | In-hospital fatal MI | 2.81 (1.40 to 5.65) | 1.28 (1.00 to 1.64) |
|  | Out-of-hospital fatal MI | 3.51 (1.90 to 6.49) | 1.52 (1.22 to 1.89) |
| Model 6 ^b^ | Total MI | 1.82 (1.60 to 2.08) | 1.17 (1.10 to 1.24) |
|  | Nonfatal MI | 1.61 (1.33 to 1.95) | 0.99 (0.91 to 1.08) |
|  | Fatal MI | 2.00 (1.69 to 2.37) | 1.33 (1.23 to 1.44) |
|  | In-hospital fatal MI | 1.81 (1.41 to 2.32) | 1.17 (1.04 to 1.31) |
|  | Out-of-hospital fatal MI | 2.12 (1.70 to 2.64) | 1.46 (1.32 to 1.61) |

MI, myocardial infarction.

^a^ The cold and heat effects were defined as the cumulative-lag risks at the 2.5th percentile (−5.2°C) and the 97.5th percentile (29.6°C) of the temperature distribution relative to the minimum morbidity temperature (24.3°C), respectively.

^b^ Model 1: unadjusted; model 2: model 1+adjusted for time and day of the week; model 3: model 2+adjusted for relative humidity and PM_2.5_; model 4: model 3+adjusted for temperature×humidity; model 5: model 3+adjusted for temperature×PM_2.5_; model 6: model 3+adjusted for humidity×PM_2.5_.

**Supplemental Table S11. Cumulative-lag relative risks and 95% confidence intervals for myocardial infarction events associated with extreme temperatures by changing the maximum lag period (4, 14, 28, and 31 days).**

| The maximum lag period | Group | Cold effects ^a^ | Heat effects ^a^ |
| --- | --- | --- | --- |
| 4 days | Total MI | 1.13 (1.06 to 1.20) | 1.15 (1.12 to 1.19) |
|  | Nonfatal MI | 1.13 (1.03 to 1.24) | 1.04 (1.00 to 1.08) |
|  | Fatal MI | 1.12 (1.03 to 1.22) | 1.25 (1.21 to 1.30) |
|  | In-hospital fatal MI | 1.02 (0.90 to 1.15) | 1.14 (1.09 to 1.20) |
|  | Out-of-hospital fatal MI | 1.20 (1.08 to 1.33) | 1.33 (1.27 to 1.39) |
| 14 days | Total MI | 1.64 (1.49 to 1.82) | 1.19 (1.14 to 1.25) |
|  | Nonfatal MI | 1.57 (1.36 to 1.82) | 1.01 (0.95 to 1.08) |
|  | Fatal MI | 1.69 (1.48 to 1.93) | 1.35 (1.28 to 1.44) |
|  | In-hospital fatal MI | 1.43 (1.18 to 1.73) | 1.18 (1.09 to 1.29) |
|  | Out-of-hospital fatal MI | 1.89 (1.60 to 2.24) | 1.48 (1.37 to 1.60) |
| 28 days | Total MI | 1.93 (1.64 to 2.27) | 1.11 (1.04 to 1.19) |
|  | Nonfatal MI | 1.65 (1.30 to 2.09) | 0.94 (0.85 to 1.05) |
|  | Fatal MI | 2.17 (1.76 to 2.69) | 1.27 (1.16 to 1.39) |
|  | In-hospital fatal MI | 1.91 (1.40 to 2.61) | 1.17 (1.02 to 1.34) |
|  | Out-of-hospital fatal MI | 2.34 (1.78 to 3.08) | 1.34 (1.19 to 1.51) |
| 31 days | Total MI | 1.83 (1.53 to 2.18) | 1.12 (1.04 to 1.21) |
|  | Nonfatal MI | 1.61 (1.25 to 2.09) | 0.94 (0.85 to 1.06) |
|  | Fatal MI | 1.99 (1.58 to 2.51) | 1.29 (1.17 to 1.42) |
|  | In-hospital fatal MI | 1.83 (1.31 to 2.57) | 1.18 (1.02 to 1.37) |
|  | Out-of-hospital fatal MI | 2.09 (1.55 to 2.82) | 1.36 (1.20 to 1.55) |

MI, myocardial infarction.

^a^ The cold and heat effects were defined as the cumulative-lag risks at the 2.5th percentile (−5.2°C) and the 97.5th percentile (29.6°C) of the temperature distribution relative to the minimum morbidity temperature (24.3°C), respectively.

**Supplemental Table S12. Cumulative-lag relative risks and 95% confidence intervals over 0 to 21 lag days for myocardial infarction events associated with extreme temperatures using different temperature cutoffs (first/99th percentiles, 5th/95th percentiles and 10th/90th percentiles).**

| Cutoffs ^a^ | Group | Cold effects ^b^ | *P* value ^c^ | Heat effects ^b^ | *P* value ^c^ |
| --- | --- | --- | --- | --- | --- |
| first/99th percentiles | Total MI | 1.99 (1.73 to 2.28) |  | 1.25 (1.16 to 1.35) |  |
|  | Nonfatal MI | 1.67 (1.37 to 2.05) | 0.016* | 1.01 (0.90 to 1.13) | 0.002* |
|  | Fatal MI | 2.25 (1.89 to 2.69) |  | 1.49 (1.35 to 1.65) |  |
|  | In-hospital fatal MI | 1.97 (1.51 to 2.55) | 0.162 | 1.26 (1.09 to 1.46) | 0.044* |
|  | Out-of-hospital fatal MI | 2.45 (1.94 to 3.08) |  | 1.67 (1.47 to 1.90) |  |
| 5th/95th percentiles | Total MI | 1.72 (1.52 to 1.96) |  | 1.12 (1.07 to 1.17) |  |
|  | Nonfatal MI | 1.56 (1.30 to 1.88) | 0.092 | 0.99 (0.92 to 1.05) | 0.002* |
|  | Fatal MI | 1.86 (1.57 to 2.19) |  | 1.24 (1.17 to 1.31) |  |
|  | In-hospital fatal MI | 1.70 (1.33 to 2.16) | 0.420 | 1.12 (1.02 to 1.22) | 0.030* |
|  | Out-of-hospital fatal MI | 1.96 (1.58 to 2.42) |  | 1.33 (1.23 to 1.43) |  |
| 10th/90th percentiles | Total MI | 1.62 (1.43 to 1.82) |  | 1.06 (1.03 to 1.09) |  |
|  | Nonfatal MI | 1.51 (1.26 to 1.80) | 0.210 | 0.98 (0.94 to 1.02) | 0.002* |
|  | Fatal MI | 1.70 (1.45 to 1.99) |  | 1.13 (1.09 to 1.17) |  |
|  | In-hospital fatal MI | 1.59 (1.26 to 2.01) | 0.656 | 1.06 (1.00 to 1.12) | 0.040* |
|  | Out-of-hospital fatal MI | 1.77 (1.44 to 2.17) |  | 1.18 (1.12 to 1.24) |  |

MI, myocardial infarction.

^a^ The first and 99th percentile of the temperature distribution was −7.3°C and 30.8°C, respectively. The 5th and 95th percentile of the temperature distribution was −3.9°C and 28.7°C, respectively. The 10th and 90th percentile of the temperature distribution was −2.0°C and 27.4°C, respectively.

^b^ The cold effects were defined as the cumulative-lag risks at the first (−7.3°C), 5th (−3.9°C), and 10th (−2.0°C) percentiles of the temperature distribution relative to the minimum morbidity temperature (24.3°C), respectively. The heat effects were defined as the cumulative-lag risks at the 99th (30.8°C), 95th (28.7°C), and 90th (27.4°C) percentiles of the temperature distribution relative to the minimum morbidity temperature (24.3°C), respectively.

^c^ *P* values were calculated by 1000 times bootstrap. Asterisks indicate *P*<0.05, suggesting a significant difference in effect sizes.

**Supplemental Table S13.** **Cumulative-lag relative risks and 95% confidence intervals over 0 to 21 lag days for myocardial infarction events associated with extreme temperatures using different reference values (25th and 75th percentiles).**

| Group | Cold effects ^a^ | Heat effects ^b^ |
| --- | --- | --- |
| Total MI | 1.24 (1.18 to 1.30) | 1.17 (1.10 to 1.24) |
| Nonfatal MI | 1.11 (1.04 to 1.19) | 0.99 (0.91 to 1.08) |
| Fatal MI | 1.34 (1.26 to 1.42) | 1.34 (1.24 to 1.44) |
| In-hospital fatal MI | 1.23 (1.13 to 1.34) | 1.17 (1.05 to 1.32) |
| Out-of-hospital fatal MI | 1.41 (1.31 to 1.52) | 1.46 (1.32 to 1.62) |

MI, myocardial infarction.

^a^ The cold effects were defined as the cumulative-lag risks at the 2.5th percentile (−5.2°C) of the temperature distribution relative to the 25th percentile (2.7°C).

^d^ The heat effects were defined as the cumulative-lag risks at the 97.5th percentile (29.6°C) of the temperature distribution relative to the 75th percentile (24.1°C).

**Supplemental Table S14. Cumulative-lag relative risks and 95% confidence intervals over 0 to 21 lag days for myocardial infarction events associated with extreme temperatures using the onset time of the last event within 28 days.**

| Group | Cold effects ^a^ | Heat effects ^a^ |
| --- | --- | --- |
| Total MI | 1.81 (1.59 to 2.07) | 1.17 (1.11 to 1.24) |
| Nonfatal MI | 1.60 (1.32 to 1.94) | 1.00 (0.92 to 1.10) |
| Fatal MI | 1.99 (1.68 to 2.35) | 1.33 (1.23 to 1.43) |
| In-hospital fatal MI | 1.80 (1.41 to 2.31) | 1.17 (1.05 to 1.31) |
| Out-of-hospital fatal MI | 2.10 (1.69 to 2.62) | 1.45 (1.31 to 1.60) |

MI, myocardial infarction.

^a^ The cold and heat effects were defined as the cumulative-lag risks at the 2.5th percentile (−5.2°C) and the 97.5th percentile (29.6°C) of the temperature distribution relative to the minimum morbidity temperature (24.3°C), respectively.

**Supplemental Table S15. Cumulative-lag relative risks and 95% confidence intervals over 0 to 21 lag days for myocardial infarction events associated with extreme temperatures changing the block length in the stationary block bootstrap method.**

|  | Group | Relative risk ^a^ | Risk difference (Block length=5) ^b^ | | | Risk difference (Block length=15) ^b^ | | |
| --- | --- | --- | --- | --- | --- | --- | --- | --- |
|  |  |  | d | 95%CI | *P* value | d | 95%CI | *P* value |
| Cold effects ^a^ | Total MI | 1.81 (1.59 to 2.06) |  |  |  |  |  |  |
|  | Nonfatal MI | 1.60 (1.32 to 1.94) | 0.09 | (−0.01 to 0.09) | 0.030* | 0.09 | (−0.01 to 0.09) | 0.048* |
|  | Fatal MI | 1.99 (1.68 to 2.35) |  |  |  |  |  |  |
|  | In-hospital fatal MI | 1.79 (1.40 to 2.29) | 0.07 | (−0.02 to 0.10) | 0.274 | 0.07 | (−0.01 to 0.10) | 0.360 |
|  | Out-of-hospital fatal MI | 2.12 (1.70 to 2.64) |  |  |  |  |  |  |
| Heat effects ^a^ | Total MI | 1.17 (1.10 to 1.24) |  |  |  |  |  |  |
|  | Nonfatal MI | 0.99 (0.91 to 1.08) | 0.13 | (0.02 to 0.08) | 0.002* | 0.13 | (0.02 to 0.09) | 0.002* |
|  | Fatal MI | 1.33 (1.24 to 1.44) |  |  |  |  |  |  |
|  | In-hospital fatal MI | 1.17 (1.05 to 1.31) | 0.10 | (0 to 0.09) | 0.024* | 0.10 | (0 to 0.09) | 0.042* |
|  | Out-of-hospital fatal MI | 1.46 (1.32 to 1.61) |  |  |  |  |  |  |

MI, myocardial infarction; CI, confidence intervals.

^a^ The cold and heat effects were defined as the cumulative-lag risks at the 2.5th percentile (−5.2°C) and the 97.5th percentile (29.6°C) of the temperature distribution relative to the minimum morbidity temperature (24.3°C), respectively.

^b^ The difference in relative risks between two groups was calculated as d=log(RR_1_)–log(RR_2_), 95% CI and *P* values were calculated by 1000 times bootstrap. Asterisks indicate *P*<0.05, suggesting a significant difference in effect sizes.

**Supplemental Table S16. Cumulative-lag relative risks and 95% confidence intervals over 0 to 21 lag days for myocardial infarction events associated with extreme temperatures stratified by subgroup (types of MI, payment methods, comorbidities, and reperfusion therapy).**

| Subgroup | | n (%) | Cold effects ^a^ | *P* value | Heat effects ^a^ | *P* value |
| --- | --- | --- | --- | --- | --- | --- |
| Type of MI | STEMI | 107,395 (49.5) | 1.35 (1.06 to 1.70) | 0.013* | 1.03 (0.93 to 1.14) | 0.539 |
|  | NSTEMI | 83,226 (38.4) | 2.14 (1.62 to 2.83) |  | 0.98 (0.87 to 1.11) |  |
| Payment methods | Urban health insurance or public reimbursement | 158,094 (72.9) | 1.55 (1.26 to 1.91) | 0.207 | 1.05 (0.96 to 1.15) | 0.096 |
|  | Other | 58,790 (27.1) | 1.98 (1.44 to 2.72) |  | 0.91 (0.79 to 1.05) |  |
| Heart failure | Yes | 79,181 (36.5) | 1.76 (1.33 to 2.33) | 0.575 | 1.04 (0.91 to 1.18) | 0.550 |
|  | No | 137,703 (63.5) | 1.59 (1.28 to 1.98) |  | 0.99 (0.90 to 1.09) |  |
| Arrhythmia | Yes | 37,203 (17.2) | 1.29 (0.87 to 1.91) | 0.181 | 1.02 (0.86 to 1.22) | 0.922 |
|  | No | 179,681 (82.8) | 1.74 (1.43 to 2.11) |  | 1.01 (0.92 to 1.10) |  |
| COPD | Yes | 7237 (3.3) | 3.18 (1.32 to 7.68) | 0.141 | 1.03 (0.67 to 1.56) | 0.929 |
|  | No | 209,647 (96.7) | 1.62 (1.35 to 1.94) |  | 1.01 (0.93 to 1.10) |  |
| Hypertension | Yes | 112,100 (51.7) | 1.75 (1.38 to 2.22) | 0.487 | 0.95 (0.85 to 1.06) | 0.101 |
|  | No | 104,784 (48.3) | 1.55 (1.21 to 1.98) |  | 1.08 (0.97 to 1.20) |  |
| Diabetes | Yes | 63,417 (29.2) | 1.74 (1.28 to 2.38) | 0.681 | 1.00 (0.87 to 1.15) | 0.906 |
|  | No | 153,467 (70.8) | 1.61 (1.32 to 1.98) |  | 1.01 (0.93 to 1.11) |  |
| Reperfusion therapy | Yes | 101,970 (47.0) | 1.39 (1.09 to 1.78) | 0.063 | 1.02 (0.92 to 1.14) | 0.800 |
|  | No | 114,914 (53.0) | 1.93 (1.51 to 2.46) |  | 1.00 (0.90 to 1.12) |  |

MI, myocardial infarction; COPD, chronic obstructive pulmonary disease; STEMI, ST-segment elevation myocardial infarction; NSTEMI, non-ST-segment elevation myocardial infarction.

^a^ The cold and heat effects were defined as the cumulative-lag risks at the 2.5th percentile (−5.2°C) and the 97.5th percentile (29.6°C) of the temperature distribution relative to the minimum morbidity temperature (24.3°C), respectively. Asterisks indicate *P*<0.05, suggesting a significant interaction.

**Supplemental Table S17. Interactive and stratified analyses of temperature and PM_2.5_ on myocardial infarction events.^a^**

| Group | Temperature | PM2.5 | | Effect of PM2.5 within  strata of temperature | RERI  (95% CI) | IRR  (95% CI) |
| --- | --- | --- | --- | --- | --- | --- |
|  |  | Low | High |  |  |  |
| Total MI | Cold | 1.00 (Reference) | 1.02 (1.01 to 1.03)* | 1.02 (1.01 to 1.03)* | −0.01 (−0.02 to 0.02) | 1.00 (0.98 to 1.02) |
|  | Heat | 1.04 (1.02 to 1.06)* | 1.06 (1.04 to 1.08)* | 1.02 (0.99 to 1.03) |  |  |
|  | Effect for temperature within  strata of PM2.5 | 1.04 (1.02 to 1.06)* | 1.04 (1.02 to 1.06)* |  |  |  |
| Nonfatal MI | Cold | 1.00 (Reference) | 1.02 (1.01 to 1.03)* | 1.02 (1.01 to 1.03)* | −0.01 (−0.03 to 0.02) | 0.99 (0.97 to 1.02) |
|  | Heat | 1.05 (1.02 to 1.08)* | 1.06 (1.03 to 1.09)* | 1.01 (0.99 to 1.03) |  |  |
|  | Effect for temperature within  strata of PM2.5 | 1.05 (1.02 to 1.08)* | 1.04 (1.02 to 1.08)* |  |  |  |
| Fatal MI | Cold | 1.00 (Reference) | 1.02 (1.01 to 1.03)* | 1.02 (1.01 to 1.03)* | 0.01 (−0.02 to 0.03) | 1.00 (0.98 to 1.03) |
|  | Heat | 1.03 (1.01 to 1.06)* | 1.06 (1.03 to 1.08)* | 1.02 (0.99 to 1.05) |  |  |
|  | Effect for temperature within  strata of PM2.5 | 1.03 (1.01 to 1.06)* | 1.04 (1.01 to 1.06)* |  |  |  |
| In-hospital fatal MI | Cold | 1.00 (Reference) | 1.04 (1.02 to 1.06)* | 1.04 (1.02 to 1.06)* | −0.03 (−0.07 to 0.01) | 0.97 (0.93 to 1.01) |
|  | Heat | 1.03 (0.99 to 1.07) | 1.03 (0.99 to 1.07) | 1.00 (0.97 to 1.03) |  |  |
|  | Effect for temperature within  strata of PM2.5 | 1.03 (0.99 to 1.07) | 1.00 (0.96 to 1.04) |  |  |  |
| Out-of-hospital fatal MI | Cold | 1.00 (Reference) | 1.01 (0.99 to 1.03) | 1.01 (0.99 to 1.03) | 0.03 (−0.01 to 0.06) | 1.03 (0.99 to 1.06) |
|  | Heat | 1.04 (1.01 to 1.07)* | 1.07 (1.04 to 1.11)* | 1.04 (1.01 to 1.07)* |  |  |
|  | Effect for temperature within  strata of PM2.5 | 1.04 (1.01 to 1.07)* | 1.06 (1.03 to 1.10)* |  |  |  |

MI, myocardial infarction; RERI, relative excess risk due to interaction; IRR, interaction relative risk.

^a^ We created categorical variables for temperature and PM_2.5_ to form an interaction term. Temperature was transformed as cold (if temperature <MMT [24.3℃]) and heat (if temperature ≥MMT), and PM_2.5_ was transformed as low (if PM_2.5_ concentration <median [62.9μg/m^3^]) and high (if PM_2.5_ concentration ≥median). Asterisks indicate *P*<0.05, suggesting a significant effect.

**Supplemental Figures**


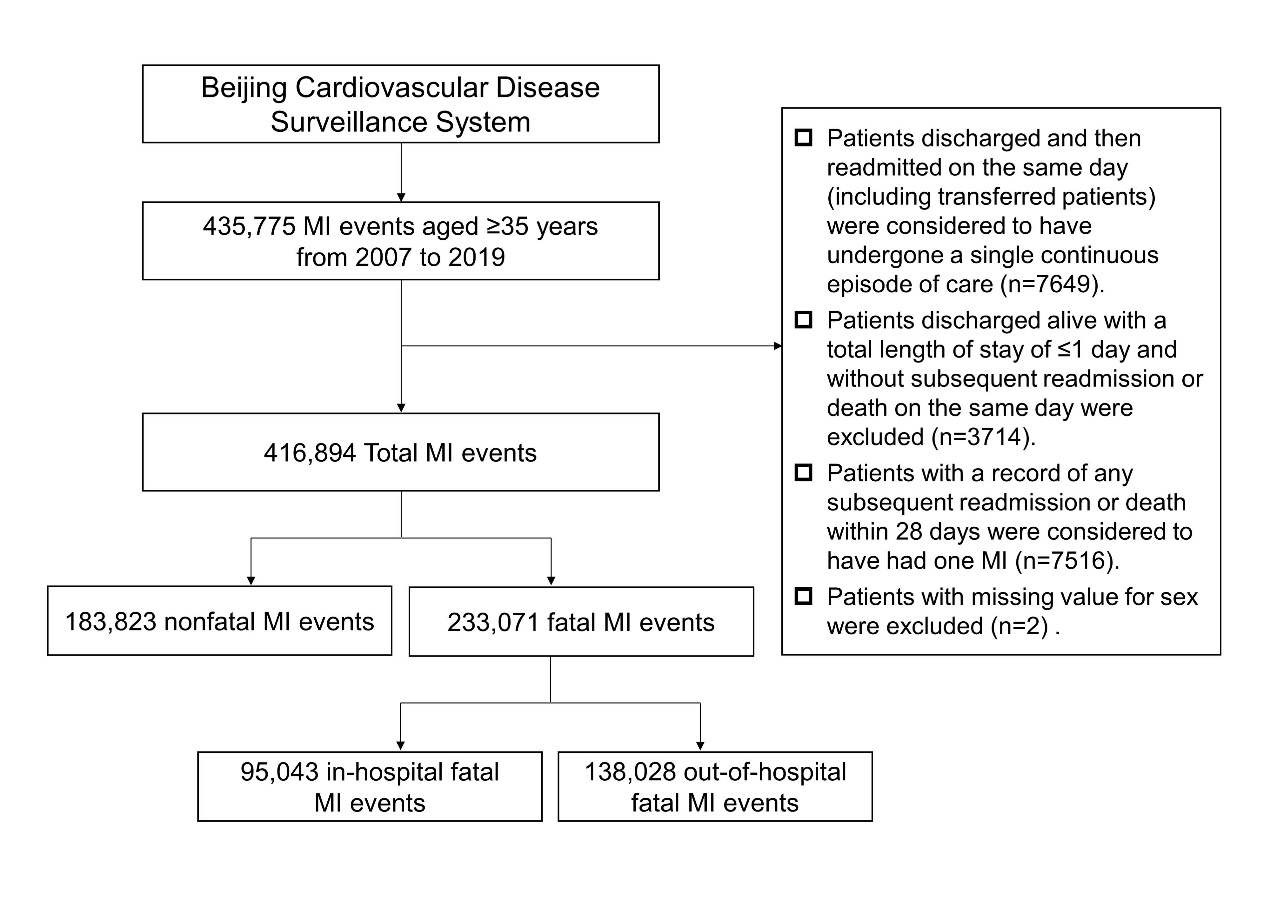


**Supplemental Figure S1. Flow diagram of myocardial infarction events enrolled in the Beijing Cardiovascular Disease Surveillance System.**

MI, myocardial infarction.


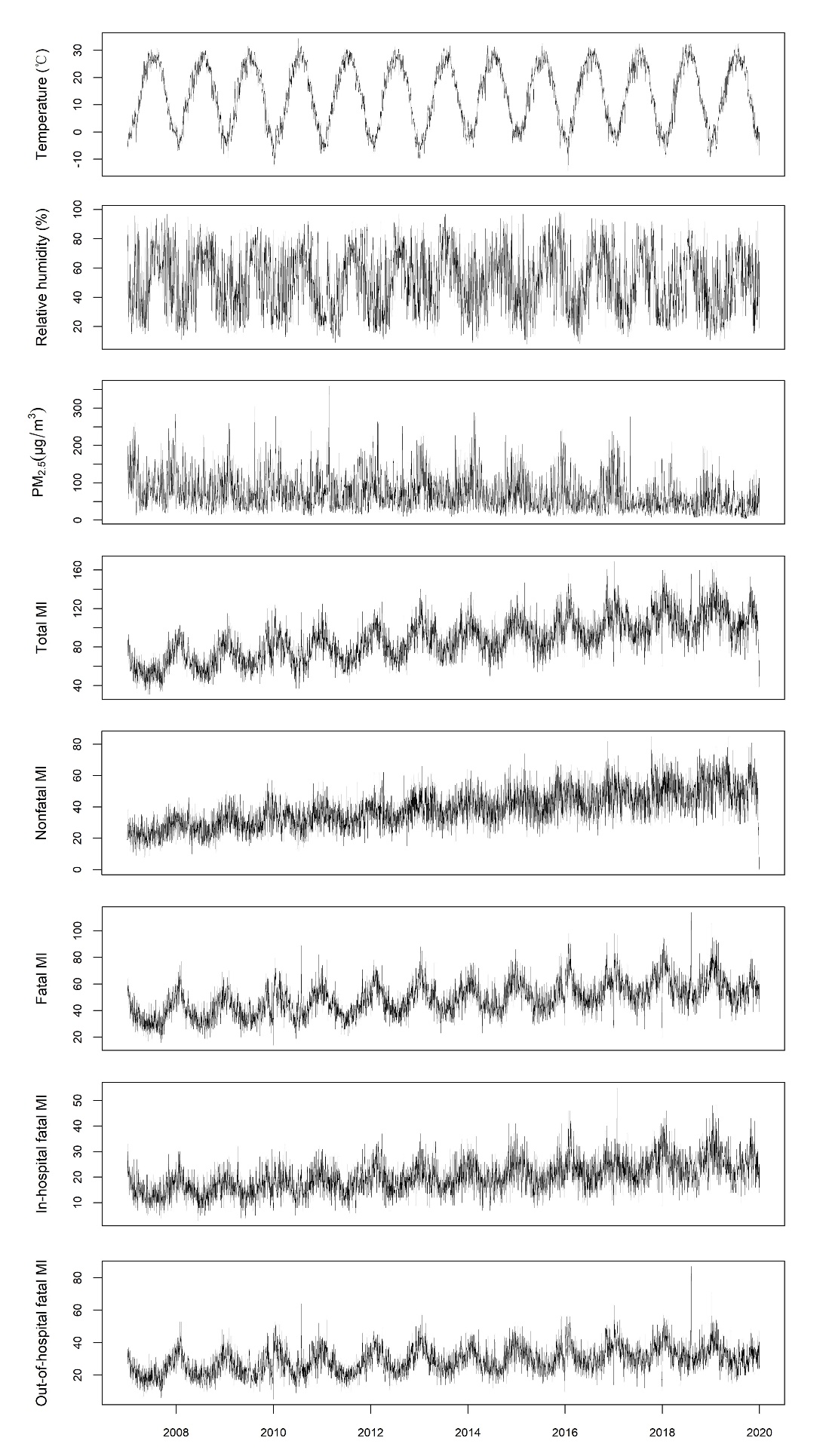


**Supplemental Figure S2. Time series plots of daily myocardial infarction events, meteorological factors, and PM_2.5_ concentrations in Beijing, China, 2007−2019.**

MI, myocardial infarction; PM_2.5_, particulate matter with aerodynamic diameter ≤2.5μm.


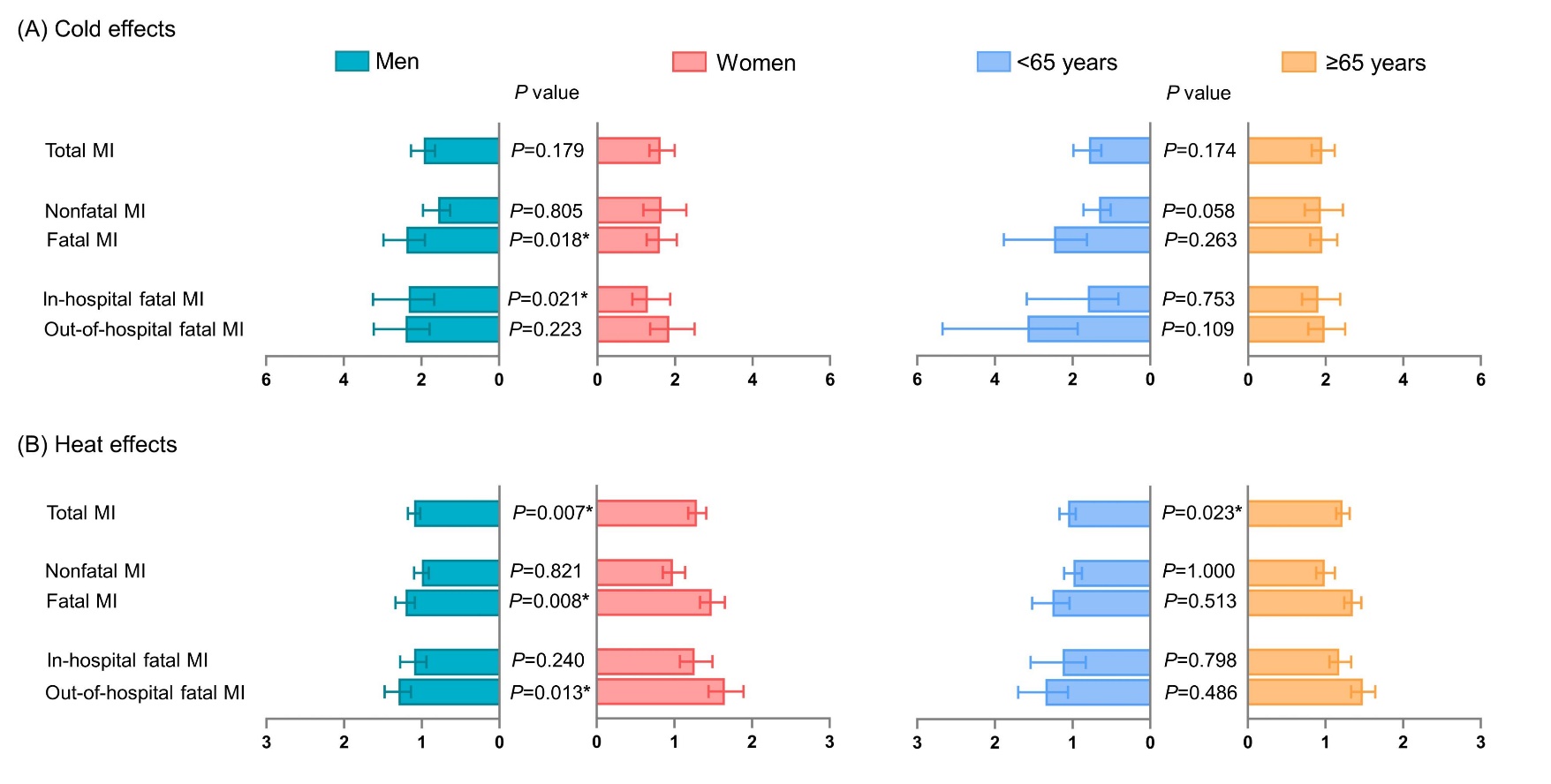


**Supplemental Figure S3. Cumulative-lag relative risks and 95% confidence intervals of cold and heat effects on myocardial infarction events over 0 to 21 lag days in sex and age groups.**

MI, myocardial infarction.

The cold and heat effects were defined as the cumulative-lag risks at the 2.5th percentile (−5.2°C) and the 97.5th percentile (29.6°C) of the temperature distribution relative to the minimum morbidity temperature (24.3°C), respectively. Green represents men and red represents women. Blue represents individuals aged <65 years and orange represents individuals aged ≥65 years. Asterisks indicate *P*<0.05, suggesting a significant interaction.


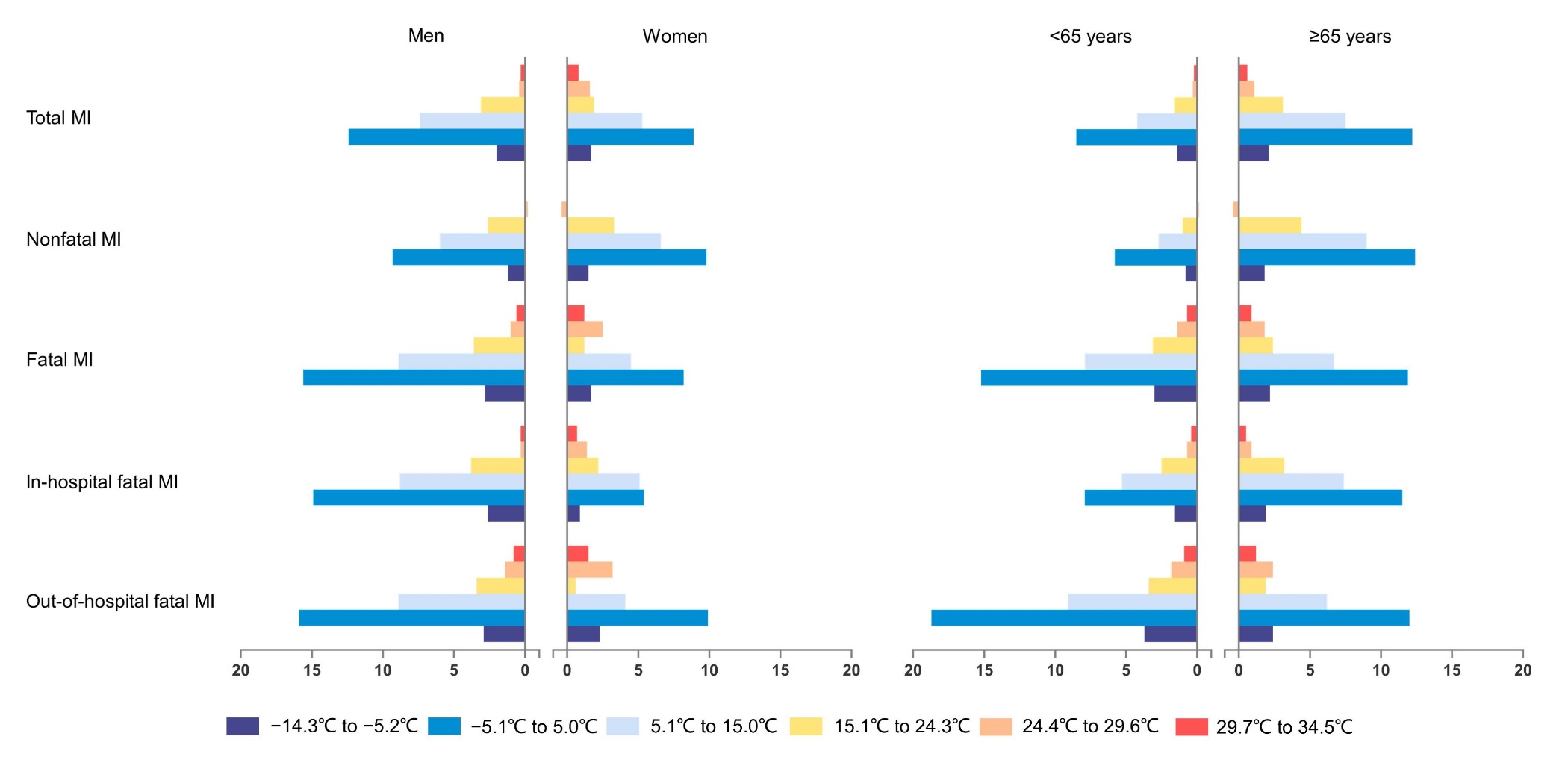


**Supplemental Figure S4. Attributable fraction (%) of myocardial infarction events attributable to nonoptimum temperatures in sex and age groups.**

MI, myocardial infarction.
